# Claudin-2 Expression Levels in Ulcerative Colitis: Development and Validation of an *In-Situ* Hybridisation Assay for Therapeutic Studies

**DOI:** 10.1371/journal.pone.0162076

**Published:** 2016-09-06

**Authors:** Kevin Randall, Neil Henderson, Jaimini Reens, Sonia Eckersley, Ann-Christin Nyström, Marie C. South, Clare A. Balendran, Gerhard Böttcher, Glen Hughes, Sally A. Price

**Affiliations:** 1 AstraZeneca R&D, Alderley Park, United Kingdom; 2 AstraZeneca R&D, Mölndal, Sweden; Universitatsklinikum Hamburg-Eppendorf, GERMANY

## Abstract

Ulcerative colitis is a chronic inflammatory disease affecting the colon and is characterized by epithelial damage and barrier dysfunction. Upregulation of the tight junction protein claudin-2 by cytokines is hypothesized to contribute to the dysregulation of the epithelial barrier. New therapeutic agents which block the action of cytokines are being investigated in patients with ulcerative colitis. In order to understand the potential of these therapies, it is important to have reliable assays that can assess downstream endpoints that reflect drug mechanism of action. The aim of the current study was therefore to establish & validate an assay to reproducibly assess the expression and distribution of claudin-2 in human colon biopsy samples. Initially, the potential to measure claudin-2 protein by immunohistochemistry (IHC) was investigated. To identify suitable reagents to develop an IHC assay, pre-established criteria were used to screen five commercial antibodies by Western blotting, immunofluorescence and immunohistochemistry on claudin-2 positive and negative cells and healthy and ulcerative colitis colon tissue. Despite some of these antibodies specifically detecting claudin-2 using some of these techniques, none of the antibodies showed the expected specific staining pattern in formalin fixed human colon samples. As an alternative method to detect claudin-2 expression and distribution in formalin fixed biopsy sections, an *in situ* hybridization assay was developed. This assay underwent a novel tiered approach of validation to establish that it was fit-for-purpose, and suitable for clinical deployment. In addition, to understand the possible relationship of claudin-2 in the context of disease severity, expression was compared to the Geboes score. Overall, the microscopical Geboes score correlated with the claudin-2 biomarker score for samples that retained crypt morphology; samples with the highest Geboes score were not specifically distinguished, probably due to crypt destruction. In summary, we have applied a strategy for identifying target-specific antibodies in formalin fixed biopsy samples and highlighted that (published) antibodies may not correctly identify the intended antigen in tissues fixed using this method. Furthermore, we have developed and, for the first time, validated an *in situ* hybridization assay for detection of claudin-2 mRNA, suitable for use as a supportative method in clinical trials. Using our validated assay, we have demonstrated that increased claudin-2 expression correlates with the severity of ulcerative colitis, where crypt destruction is not seen.

## Introduction

Ulcerative colitis (UC) and Crohns Disease (CD) are chronic inflammatory bowel diseases (IBD). Ulcerative Colitis affects the colon and is morphologically characterized by inflammation, epithelial damage and crypt erosions/ulcerations. In UC, the aetiology and pathogenesis is not known, but a combination of genetic and environmental factors are thought to result in gut wall inflammation and epithelial barrier dysfunction. This dysfunction may lead to increased membrane permeability, allowing leaking and enabling the luminal contents to be subjected to the mucosal immune system. Epithelial barrier dysfunction may be mediated, at least in part, by anti-inflammatory Th2 cytokines including IL-13. IL-13 producing cells are present in healthy colonic mucosa, where IL-13 is thought to play a role in the defence from normal gut microbial pathogens. However, in UC patients, IL-13 production by lamina propria lymphocytes is significantly elevated compared to control patients or patients with Crohn’s ileocolonic inflammatory disease [1], [2].

The intestinal epithelial barrier is maintained by tight junctions at the apical surface, comprised of a complex of proteins including transcellular filament proteins, scaffold proteins and members of the claudin family, including claudin-2. Tight junctions maintain polarity of cells by preventing lateral diffusion of proteins between apical and basolateral membranes and prevent the paracellular transport of molecules and ions. Claudin-2 forms high conductance, paracellular cation-selective pores [3], which determine the paracellular ion selectivity and water permeability [4]. Claudin-2 has been reported to be undetectable in normal human colon samples in some studies [5], [6], [7], to show restricted expression in undifferentiated crypt cells [8] or to be expressed in both mucosal epithelium and crypts [9], [10]. In inflammatory bowel diseases, including active ulcerative colitis, there is an up-regulation of claudin-2 protein [6], [11], [10], accompanied by structural changes in the tight junctions; together these may be responsible for the loss of selectivity of tight junctions in patients with inflammatory bowel diseases. Increased expression of claudin-2 is likely to be downstream of IL-13 mediated STAT6 activation [2], [12].

Currently, diagnosis and assessment of disease severity of inflammatory bowel diseases, such as UC, are usually based on a combination of clinical, radiological, endoscopic, and microscopic criteria [13]. Different histological scoring systems have been designed to assess microscopic mucosal disease activity and have been used widely in clinical drug trials, usually assessing chronic and acute changes including structural, epithelial and inflammatory features. Two widely used microscopic scoring systems are the Riley score [14], which gives a combined score from individual histological features, and the Geboes score [13] which partly overlaps with the Riley score components (e.g. acute and chronic inflammatory cell infiltrate, crypt abscesses, surface epithelial integrity, and crypt architectural irregularities). Other scoring systems have also been used that are modifications of the Riley score (detailed review by [15]).

In seeking to discover new therapeutic treatments, biomarkers have become an integrated part of drug development and clinical practice, with regulatory agencies encouraging their use [16]; [17]. In clinical trials, establishing that a drug molecule engages with its biological target, (proof-of-mechanism, PoM), is a critical step in demonstrating appropriate pharmacology. PoM studies rely on the identification of target associated mechanism-of-action biomarkers. Since claudin-2 is elevated in UC patients, we hypothesized that it could be utilized as a PoM biomarker in the development of novel UC drug therapies, provided a validated quantitative assay was available. The aim of the current study was to establish and validate a biomarker assay for claudin-2, suitable for use ultimately on formalin fixed paraffin embedded (FFPE) sections of routine human colon biopsies. In addition, we used our validated methodology to determine the relationship between claudin-2 expression and microscopical disease activity measurements (e.g. Geboes score).

## Materials and Methods

### Tissue collection

All clinical study patients (132 samples from 78 individuals) provided written informed consent and the multi-centre clinical study was conducted in accordance with local ethical guidelines and with the UK Human Tissue Act (2004) and Declaration of Helsinki. Local Review Boards included: NRES Committee South Central-Southampton B (ref 12/SC/0119, 15 Aug 2012); Comite de Protection des Personnes Nord-Ouest II (ref 2012/3, 24 Sept 2012) and Comitao Etico per la Sperimentazione Clinica dei Medicinali dell Azienda Ospedaliero-Universitario Careggi di Firenze (ref 696/11, 16 Apr 2012). Endoscopically guided forceps were used to collect biopsy tissue following routine clinical procedures (56 biopsies were classified as “Unaffected/normal”, and 76 biopsies were classified as “Affected”). Samples were fixed within 20 minutes of excision, using 10% neutral buffered formalin solution at room temperature for 24–48 hours, before processing into wax. Biopsies were aligned to allow analysis of a transverse section of representative colon mucosa.

Commercial biopsy material, purchased from Adeptbio, Indivumed or Tristar suppliers, was obtained endoscopically or from ressected biopsy tissue, and processed as described above. Commercial materials were collected in accordance with ethical guidelines and relevant ethics committees including Hamburg Chamber of Physicians/Georgetown University, USA (Indivimed), University of Minnesota Clinical and Translational Science Institute, funded by NIH Clinical and Translational Science Award UL1TR000114, and the University of Minnesota Academic Health Center (Adeptbio), Ethical Committee of the Hospital General Universitario Gregorio Maranon (Tristar).

No patient/donor identifying information was given to the authors. To avoid bias during scoring process, information on activity status (“Affected” or “Unaffected/normal”) of clinical study samples or commercial biopsy material, as assigned by the endoscopist, was not divulged to the claudin-2 scoring-pathologist. Samples were unblinded post-analysis, by the primary authors, to assess any correlation between activity score (as assigned by pathologist) or Geboes score (as assigned by endoscopist) against claudin-2 score (as assigned by pathologist) in both the validation and the clinical study bioanalysis respectively.

### Antibodies

Five commercially available, and well documented, anti-claudin-2 antibodies were identified for screening by Western blotting, immunocytochemistry and immunohistochemistry: a rabbit polyclonal raised against the N-terminus of claudin-2 (AP23596PU-N, Acris Antibodies); three rabbit polyclonals raised against the C-terminus (51–6100, Life Technologies, IMG-80487, IMGENEX and NBP1-67516, Novus Biologicals) and a mouse monoclonal (12H2) raised against 26 amino acids within the C-terminus of claudin-2 (32–5600, Invitrogen).

### Cell lines

CHO-K1, Chinese Hamster Ovary cells, originally isolated by Puck et al. (ATCC^®^ CCL-61^™^) and colon epithelial T84 cells, deposited by Masui, H, (ATCC^®^ CCL-248^™^) were obtained from the American Type Culture Collection (ATCC) and cultured in Ham’s F12/10% foetal bovine serum (FBS) or Dulbecco’s Modified Eagle Medium (DMEM)/Ham’s F12 (1:1), 5% FBS respectively [18,19]. HT29 colon adenocarcinoma cells, were provided by the European Collection of Cell Cultures (ECACC 85061109), deposited by Harris, H and Sutherland, R [20]. These were cultured in Eagle's Minimum Essential Medium (EMEM)/10% FBS/1% non essential amino acids (NEAA).

### Generation of green fluorescent protein tagged claudin-2 (GFP-CLDN2) overexpressing CHO-K1 cells

CHO-K1 cells were cultured to approximately 50% confluency, in T175 flasks, prior to transiently transfecting with human claudin-2 cDNA ORF clone (RG204199, Origene) using Lipofectamine 2000 (11668027, Invitrogen) according to the manufacturer’s instructions. No media change was carried out and cells were harvested after approximately 48 hours. Untreated CHO-K1 cells were simultaneously set up as a negative control.

### Cell lysate preparation

One T175 of each cell line was grown to approximately 70% confluency, washed twice in phosphate buffered saline (PBS) and cells harvested with 5 mL of 0.25% trypsin/1 mM EDTA (25300, Life Technologies). Cells were resuspended in growth media prior to centrifugation for 5 minutes at 300G. The cell pellet was washed twice in ice cold PBS prior to lysis in ice-cold RIPA buffer (50 mM Tris-HCl, pH 7.4, 150 mM NaCl, 1% NP40, 0.25% sodium deoxycholate, 1 mM PMSF, protease inhibitor cocktail (04693159001, Roche) and phosphatase inhibitor cocktail (78420, Pierce). The sample was immediately frozen on dry ice and stored at -80°C.

### Western blot analysis

40 μg of each cell lysate and 40 ng recombinant human GST tagged claudin-2 (H00009075-P01, Abnova) were loaded onto 4–12% Bis-Tris gels (345–0123, Biorad) and electrophoresed for 55 minutes at 200V in MOPS buffer. Protein was transferred onto nitrocellulose membrane for 1 hour at 100V in Towbin buffer plus 0.01% SDS. A section of membrane was stained with Ponceau-S to confirm protein transfer had taken place. Non-specific protein binding was blocked with 4% (w/v) Marvel milk powder/PBST for 1 hour at room temperature with rocking before incubating with primary antibodies (1:500) in 2% (w/v) Marvel/PBST overnight at 4°C. Blots were washed 3 times for 10 minutes in PBST prior to incubation with appropriate species specific secondary antibodies: goat anti-rabbit-HRP (A9169, Sigma) used at a dilution of 1:5000 or rabbit anti-mouse-HRP (P0260, DAKO) used at a dilution of 1:2500, both diluted in 2% (w/v) Marvel milk powder/PBST. Secondary antibody only control blots were used alongside an anti-GAPDH antibody (ab37168, Abcam) as a loading control. After a 1 hour incubation with secondary antibody, membranes were washed as before, prior to developing using enhanced chemiluminescence (ECL) reagent (RPN2106, GE Healthcare) and images taken on Fujifilm camera.

### Immunofluorescence imaging

Cells were seeded at a density of 1x10^5^ cells/ml in 96 well plates (3904, Costar) and grown at 37°C, 5% CO_2_ until around 70% confluence. CHO-K1 cells were transiently transfected with GFP-claudin-2 as described earlier. Cells were fixed for 20 minutes at room temperature in 10% neutral buffered formalin solution before being thoroughly washed in PBS. Then cells were stored at 4°C until required for staining.

For immunofluorescence, cells were permeabilised with 0.1% Triton/PBS for 10 minutes. Primary antibodies were applied at a dilution 1:500 for 1 hour at room temperature, before washing in PBS/0.1% Triton-X100. Appropriate secondary antibodies were applied at a dilution of 1:500 for 1 hour at room temperature (donkey anti-rabbit pAb IgG DyLight 594nm (ab96921, Abcam) or goat anti mouse IgG AlexaFluor 546 (A11030, Life Technologies)). Cells were washed twice in PBST, and once in PBS before counterstaining with Hoechst 33342 (H3570, Life Technologies) at a dilution of 1:5000 in PBS for 10 minutes. After 2 further PBS washes, images were read on an ImageXpress system (Molecular Devices, US).

### Immunoprecipitation

One T175 flask of T84 cells was grown to 70% confluence, washed twice in PBS and harvested in trypsin/EDTA, before resuspension in growth media. Cells were pelleted by centrifugation for 5 minutes at 300G and washed twice in PBS before lysing in ice cold immunoprecipitation lysis buffer (150 mM NaCl, 50 mM Tris, pH7.5, 5% Glycerol, 1% NP-40, 0.1% sodium-deoxycholate, 1 mM MgCl_2_, protease inhibitor cocktail (04693159001, Roche) and phosphatase inhibitor cocktail (78420, Pierce). The sample was frozen, thawed on ice and aliquoted into 2 equal volumes and probed with 10 μg primary antibody alongside non-specific IgG control antibody, with rocking for 45 minutes at 4°C. Immunoprecipitation was then performed according to the manufacturer’s instructions. Briefly, 50 μL μMACS Protein A or G MicroBeads (130-071-001/101, Miltenyi Biotec) were added to each sample and incubated for a further 45 minutes before loading twice onto a μMACS column, pre-equilibrated in wash buffer (150 mM NaCl, 50 mM Tris, pH 7.5, 1% NP-40). The column was washed twice before elution into pre-heated (95°C) reducing Laemmli buffer (161–0737, Biorad).

40 μL of eluted immunoprecipitation sample was loaded onto 4–12% Bis-Tris criterion gels and electrophoresed for 55 minutes at 200V in MOPS buffer. The gel was then stained in Imperial Protein Stain (24615, Biorad), and washed in reverse osmosis H_2_O before isolation of bands for mass spectrometry analysis. Samples were also used for Western blotting (as previously described) and probed to check for bands cross-reacting with two Western blot compatible anti-claudin-2 specific antibodies (32–5600 & NBP1-67516).

### Liquid chromatography-mass spectroscopy (LC-MS)

The band of interest was excised from the gel and digested using 15 μL of 5 ng/μL trypsin (V5111, Promega) in 25 mM ammonium bicarbonate overnight at 37°C. The peptide digest was extracted from the gel with 5% acetonitrile (ACN), 2% formic acid (FA) in water. Then 8 μL of the extract was subjected to LC-MS/MS analysis on a 1200 HPLC-Chip system coupled with the 6520 Accurate-Mass Q-TOF LC-MS (Agilent Technologies, California, US). A HPLC-Chip with ZORBAX 300SB-C18 (300Å), 5 μm particles, 40 nL enrichment column, and a 75 μm x 43 mm analytical column was used. Mobile phases were mixtures of ACN, water and FA. A 20 minute linear gradient from 5–40% ACN was used for the separation on the analytical column. The MS/MS spectra were converted to peak lists in the Mascot generic format (mgf) and used in a SwissProt database search with MS/MS Ion Search (Mascot, Matrix Sciences). Database search parameters included: enzyme: trypsin, variable modification: oxidation of methionine, peptide mass tolerance: ± 10 ppm and fragment mass tolerance: ± 0.3 Da.

### Preparation of formalin fixed paraffin embedded (FFPE) cell pellets

Three T175 flasks of each cell line (HT29 cells for positive control and CHO K1 cells for the negative control) were grown to around 70% confluency, washed twice in PBS and harvested by directly scraping cells into 10% neutral buffered formalin. Cells were fixed overnight at 4°C, before being spun down and washed twice PBS, resuspended in 3% low melting-point agarose in PBS and assembled into an embedding cassette. The sample was stored in 80% ethanol at room temperature prior to embedding in wax.

### Claudin-2 immunocytochemistry and immunohistochemistry

The specificity of antibodies was initially screened using FFPE claudin-2 over expressing and negatively expressing CHO-K1 cell pellets. HT-29 cells endogenously expressing claudin-2 were also used as a positive control. Each anti-claudin-2 antibody underwent a method development procedure for antigen retrieval using: no pre-treatment, 1 mM EDTA 110°C for 2 minutes, 10 mM citrate 110°C for 2 minutes or Proteinase-K (S3020, Dako Ltd, Cambridge, UK) for 2 minutes. The antigen retrieval method and antibody concentration resulting in optimal staining for each anti-claudin-2 antibody was taken forward to apply on the colon tissue samples.

For immunohistochemistry, 4 μm thick human colon sections were dewaxed in xylene and rehydrated through graded alcohols to water. Tissue sections were placed onto a Labvision Immunostainer (Thermo Fisher Scientific, UK) for immunohistochemical staining. Sections were first washed in Tris buffered saline/0.1% Tween (TBST) followed by blocking of endogenous peroxidase with 3% hydrogen peroxide/TBST for 10 minutes. After a buffer wash, non-specific Ig-binding sites were blocked for 20 minutes using a background blocker with casein (MP-966-P100, A Menarini Diagnostics Ltd, Berkshire, UK). This was followed by application of primary antibody, detection with the appropriate secondary antibody, either a mouse (K4001, mouse Envision System-HRP Labelled Polymer, Dako UK Ltd) or rabbit EnVision-HRP, (MP-XCP-U100, X-Cell-Plus HRP Detection Kit, A Menarini Diagnostics Ltd) and visualisation using 3,3’-diaminobenzidine (DAB) (Menarini Diagnostics) for 10 minutes. Following a water wash, sections were counterstained in Carazzi’s haematoxylin for 1 minute and mounted non-aqueously. Negative controls were performed using either an isotype immunoglobulin or by omitting primary antibody. Claudin-2 expressing cell lines (see above) were used as positive controls. All dilutions were in TBST and procedures performed at room temperature.

### Claudin-2 in-Situ hybridisation (ISH)

Sequential consecutive (adjacent) or non-consecutive (non-adjacent) 4 μm sections of FFPE biopsy samples were pre-treated by heating to 60°C for 30 minutes, followed by incubation for 60 minutes at room temperature in 10% formaldehyde (in PBS), and de-waxed using xylene (twice) and 100% ethanol. Slides were air-dried and stored overnight for up to 4 days. At this stage, slides were classified as “pre-treated”. Samples were then retrieved through boiling in pre-treatment solution from the QuantiGene ViewRNA ISH Tissue Assay kit (QVT-0051, Panomics) for 5 minutes (cells) or 10 minutes (colon tissue), with subsequent incubation with kit protease for 5 minutes at 38.5°C, and subsequent 4% formaldehyde/PBS post-fixation at room temperature for 5 minutes. Slides were then incubated with relevant QuantiGene ViewRNA probe set for either claudin-2 or a sense probe as negative control (VA1-12003-01 and VA1-14363-01, Panomics) for 2.5 hours at 38.5°C to allow hybridization with the mRNA. Slides were then treated with PreAmplifier/Amplifier solutions to enhance signal prior to incubation with a LP1-AP reporter probe. Finally, the LP1-AP reporter probe was visualized with Fast Red Substrate and the slides counterstained/mounted with aqueous mountant. The intensity and distribution of positive stain was then assessed and scored. Non-adjacent tissue slides had at least a 24 μm gap between sections. Adjacent tissue slides had 0–4 μm gap between sections.

### Scoring criteria for ISH on colon tissue

For the validation, all ISH and H&E stained tissue sections were assessed by a single pathologist. The scoring criteria was a semi-quantitative assessment (grades 0 to 5, where 0 was none, 1 was minimal, 2 was slight, 3 was moderate, 4 was marked and 5 was severe), considering the overall amount of stain in terms of both intensity and distribution over relevant microscopic mucosal structures. The underlying observational assessments of distribution and localisation of stain were recorded separately as qualifiers to the stain intensity e.g. focal versus multi-focal or diffuse, and crypt-based versus surface epithelial localisation. For evaluation of clinical samples, ISH staining for claudin-2 was assessed by a single pathologist, and Geboes score by an independent pathologist.

### Scoring criteria for ISH on cell pellet sections and image analysis

Formalin fixed cell pellet blocks were used for quality control of each experiment. The amount of staining in cell controls was visually assessed (using a 0 to 5 scoring system) by a single analyst and also quantified using an image analysis algorithm. Briefly, a representative area of the slides was digitised at 40x magnification using an Aperio ScanScope AT scanner (Aperio, USA). Manual focussing of the instrument was sometimes necessary to ensure the ISH signal was captured correctly. The images were analysed using a bespoke rule set within the Tissue Studio 3 application (Definiens AG, Germany), optimised to identify cells on the slide and, within those cells, measure the number of red pixels (ISH signal) and the number of blue pixels (haematoxylin counterstain). The output was a positive pixel percentage for each slide, based on the calculation:

Positivepixelpercentage(%)=(No.ofredpixels/(No.ofredpixelsplusbluepixels))x100

### Statistics

The replicate results generated to explore different aspects of repeatability and reproducibility of the claudin-2 ISH method are all presented graphically. Individual results, together with means and ranges where appropriate, are provided. Goodman and Kruskal’s Gamma [21], a measure of association between ordinal variables, was used to assess the degree of concordance between the Geboes scores and the claudin-2 ISH scores for 132 samples with both scores available.

## Results

### Claudin-2 antibody validation strategy

There is a growing awareness within the literature of the lack of data accompanying commercially available antibodies, and the subsequent need to validate antibodies in-house. In agreement with a number of papers published, an antibody validation strategy was formulated to determine the selectivity, specificity and IHC compatibility of the selected antibodies [22],[23], [24]].

### Anti-claudin-2 antibody selection

**Fig 1:**
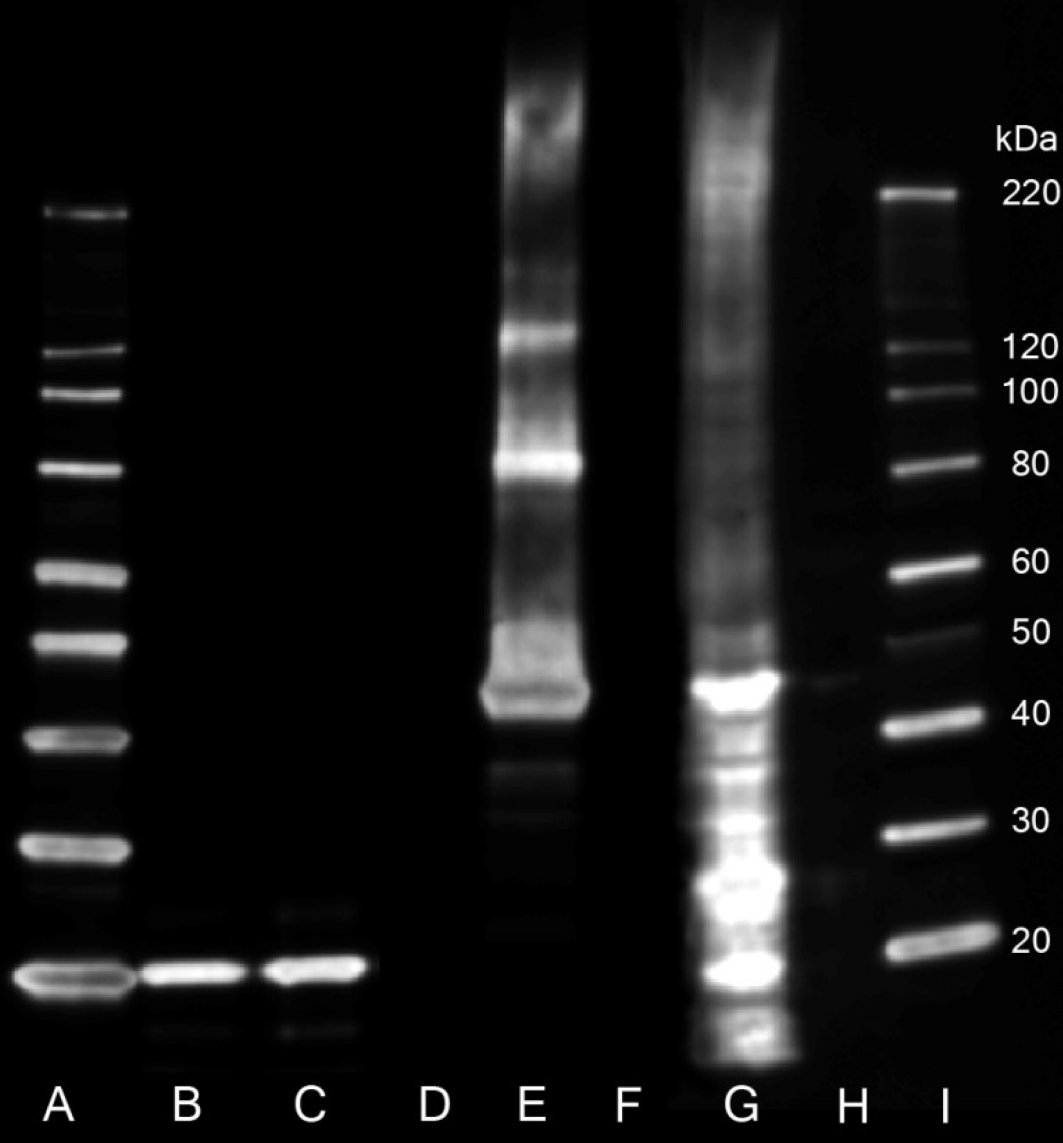
Western blot analysis to demonstrate the specificity of the 32–5600 (12H2) antibody to recognise Claudin-2 protein transcript. 40 **μ**g of each cell lysate and 40 ng recombinant human GST tagged claudin-2 was loaded. Lane order is as follows: (A & I) magic marker, (B) HT29 cell lysate, (C) T84 cell lysate, (D & F) empty lane, (E) recombinant GST-Claudin-2 protein, (G) CHO K1 (overexpressing GFP-CLDN2 construct) cell lysate and (H) CHO K1 (untransfected control) cell lysate. 12H2 clearly detects recombinant GST-CLDN2 protein (lane E, 52kDa). CHO-K1 cells do not express CLDN2, as seen in lane H, however upon transient transfection of CHO-K1 cells with a GFP-CLDN2 construct (53kDa), the antibody detects the overexpressed construct. 12H2 is able to detect endogenous levels of Claudin-2 protein (MW ~20kDa) in the HT29 & T84 cell lysates (B & C respectively), as confirmed by mass spectrometry (S2 Fig).

Out of the 5 antibodies investigated, only antibody 12H2 showed the desired profile in all applications tested (Table 1). In Western analysis (Fig 1), 12H2 recognised the recombinant GST-claudin-2 protein (51kDa); the negative cell line CHO K1 was completely clear unless cells were transfected with GFP-claudin-2 and distinct clean bands of ~20kDa were seen in the endogenously expressing T84 and HT29 cells lines, as is well documented in literature [25,26]. Molecular weight interpretation of a protein via Western blot will only ever be an estimation at best, however as endogenous Claudin-2 is 24.5kDa, the reported difference in band size was investigated further to ensure this signal was not off-target binding.

**Table 1:** Outcome of the five commercial antibodies in the screening process.

Antibody	Successful in	Failed in	Outcome
IMG-80487	WB, IF(CHO)	ICC	Deselected
AP23596PU-N		WB, IF (CHO&HT29)	Deselected
NBP1-67516	WB, IF(CHO)	ICC	Deselected
51–6100		WB, ICC	Deselected
32–5600	WB, IF (CHO & HT29), ICC, IP/MS	IHC(NSB*)	Lead antibody

Outcome was selected upon the antibody performance following analysis by Western blotting (WB), Immunofluorescence in CHO or HT29 cells (IF), Immunocytochemistry (ICC), Immunoprecipitation with Mass spectrometry (IP/MS) or Immunohistochemistry (IHC).

NSB* indicates observed non-specific binding of the antibody

Immunoprecipitation analysis of 12H2 immunoprecipitated T84 cell lysate was carried out, run out on SDS-PAGE, the 20kDa band excised, from which Claudin-2 specific peptides were successfully identified by mass spectrometry, suggesting that 12H2 recognises the claudin-2 epitope by Western blot, as well as in solution (S2 Fig).

**Fig 2:**
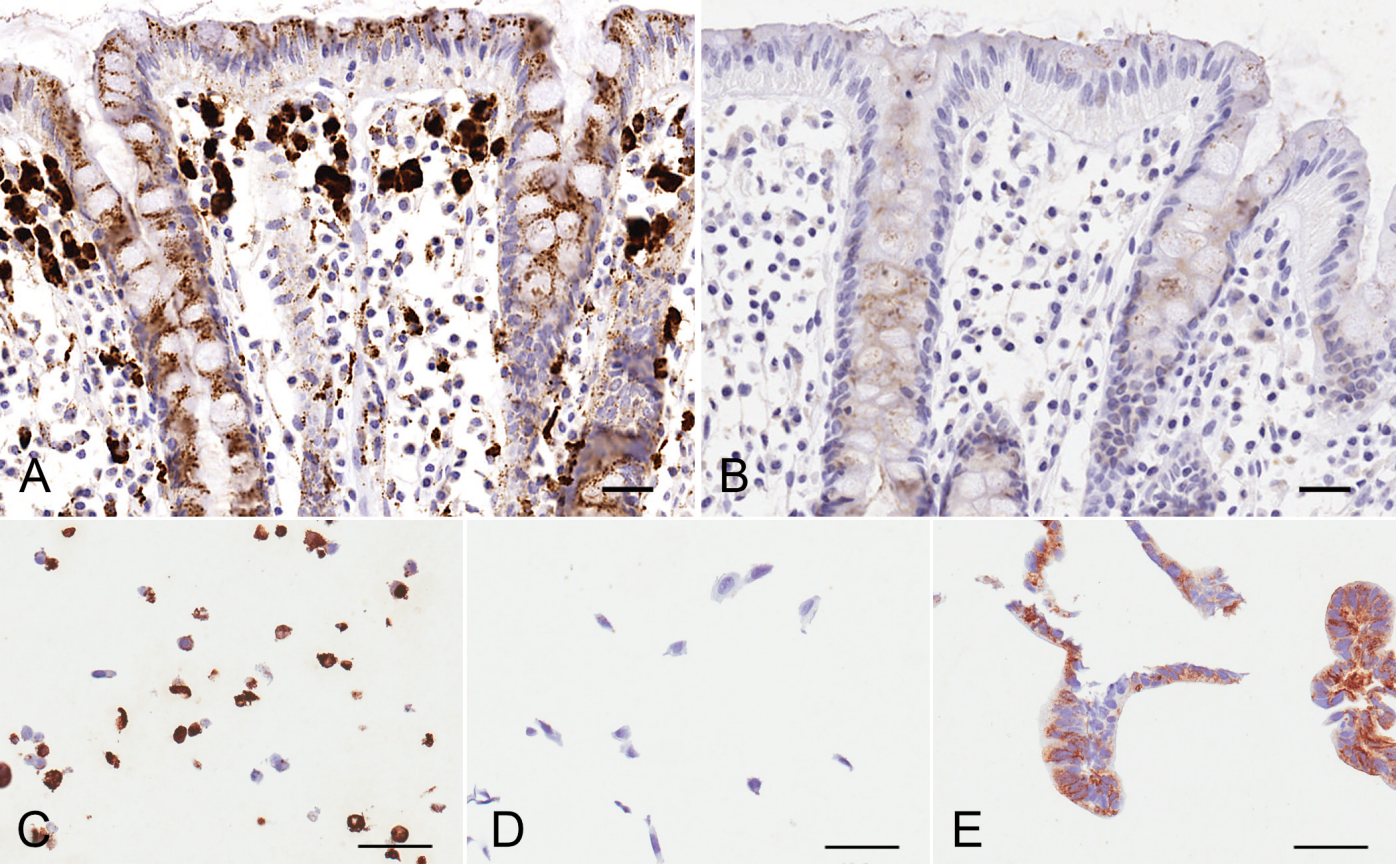
Lead candidate antibody staining patterns for claudin-2 in cell models. Fig 2 shows claudin-2 staining, using (A) the lead candidate antibody (12H2) or (B) isotype control in diseased UC. Panels (C-E) show the claudin-2 staining patterns in ICC FFPE cell pellets using 12H2. Specifically, (C) CLDN2 overexpressing CHO K1 cells, (D) untransfected CHO K1 cells and (E) T84 cells. The scale bars represent 50 **μm**.

This antibody was compatible in immunofluorescence and produced distinct membrane staining in endogenously expressing HT29 (S1 Fig). No staining was seen in the CHO-K1 negative cell lines. Upon transient transfection of GFP-claudin-2 in CHO-K1 cells, 12H2 was able to show clear co-localisation of staining suggesting specific binding in this application. This staining profile was also reproducible in immunocytochemistry against FFPE HT29, T84, CHO-K1 and overexpressing GFP-claudin-2 CHO K1 cells (Fig 2C–2E). We therefore concluded that the Invitrogen anti-claudin-2 antibody (12H2) was the only antibody suitable to take forward for immunohistochemistry evaluation on FFPE colon tissue.

Immunohistochemistry with 12H2 on FFPE normal human colon tissue sections showed punctate staining between epithelial cells within the crypts and also in the surface epithelium (Fig 2A). Strong staining was also observed in the sub-apical cytoplasm of the epithelial cells. This localization was not the typical “chicken wire” pattern seen with other proteins that locate to tight junctions. Strongly positive immunoreactive cells were also observed in the lamina propria immediately underlying the surface epithelia, most likely macrophages. Some cells deeper in the lamina propria were also strongly positive but these were more sparsely distributed. Sections of human colon stained with isotype matched IgG control (IgG2b) showed only very weak diffuse staining, consistent with non-specific detection of the mucus lining (Fig 2B).

Several studies suggest that there is very low or no expression of claudin-2 in normal human colon [5], [6], [7]. We therefore optimized 12H2 on colon samples from patients with ulcerative colitis to determine whether these samples would show a more expected distribution of positive signal. Several different samples were used but a similar pattern of staining was obtained in samples from diseased colon compared with normal colon. Furthermore, the signal intensity with 12H2 varied considerably between different samples. In these samples, the signal intensity did not correlate with disease activity; both control and diseased samples were demonstrated to show high and low immunoreactivity (data not shown).

To understand the protein expression pattern seen with 12H2, we carried out ISH to determine whether claudin-2 immunopositive cells also expressed claudin-2 mRNA. mRNA probe specificity was tested in a similar manner to the screening of antibodies, using endogenously expressing cells and positively/negatively transfected cells.

**Fig 3:**
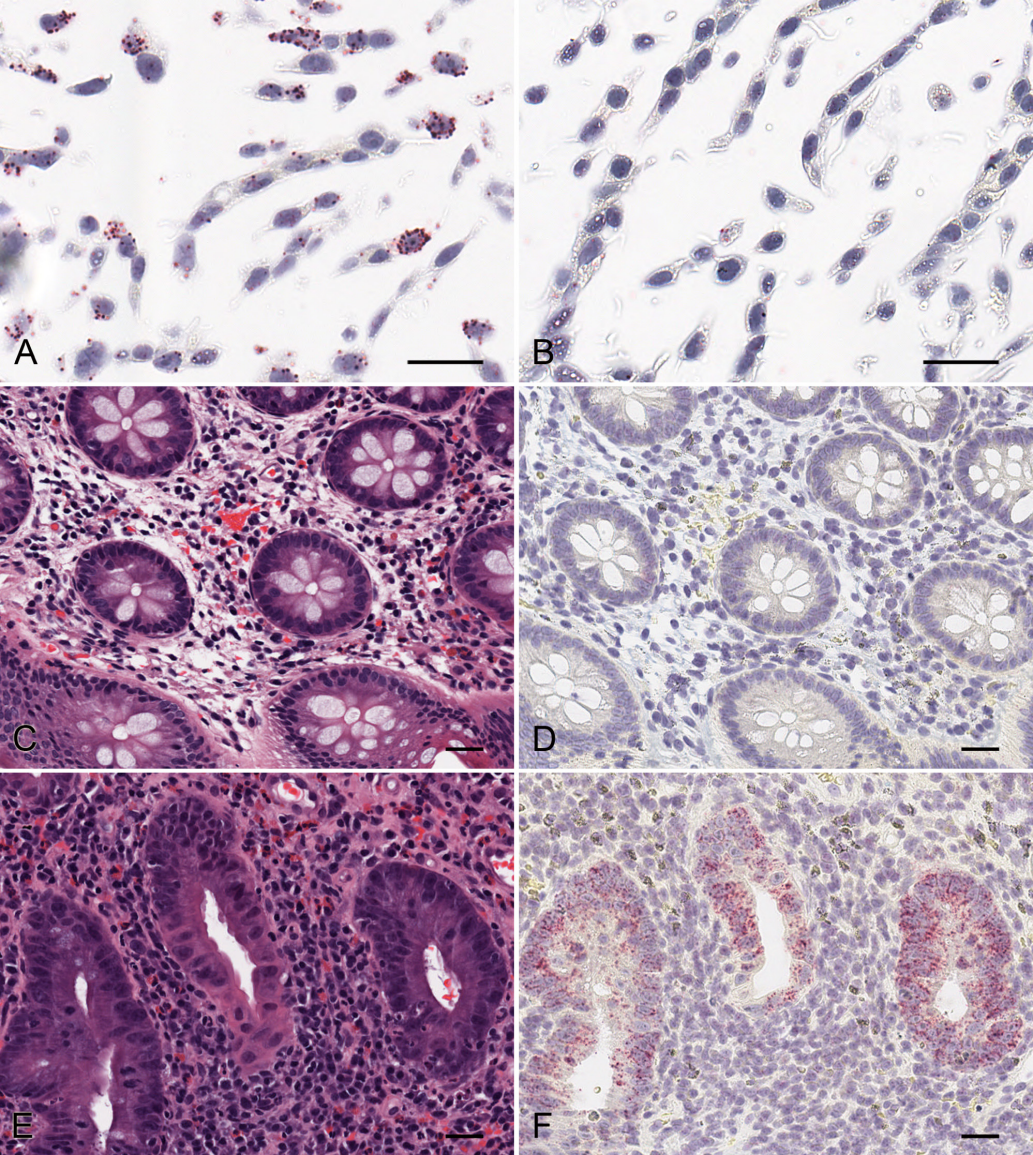
Comparison of claudin-2 ISH signal in affected and unaffected UC colon biopsies. Fig 3 shows *in situ* hybridisation (ISH) staining in CLDN 2 overexpressing CHO K1 cells using QuantiGene ViewRNA (A) claudin-2 probe (VA1-12003-01) and (B) SENSE probe (VA1-14363-01) from Panomics. Adjacent sections from unaffected UC colon biopsy material were H&E stained (C) or claudin-2 ISH stained (D); adjacent sections from affected UC colon biopsy material were H&E stained (E) or claudin-2 ISH stained (F). The scale bars represent 50 μm.

ISH with the claudin-2 probe showed a strong positive signal in HT29 cells (endogenously expressing claudin-2) and no staining in the negative control (sense probe; Fig 3A and 3B). The positive cells were comparable in number and morphology to those that stained positive by immunohistochemistry with 12H2 (data not shown).

### Validation of an ISH method

**Fig 4:**
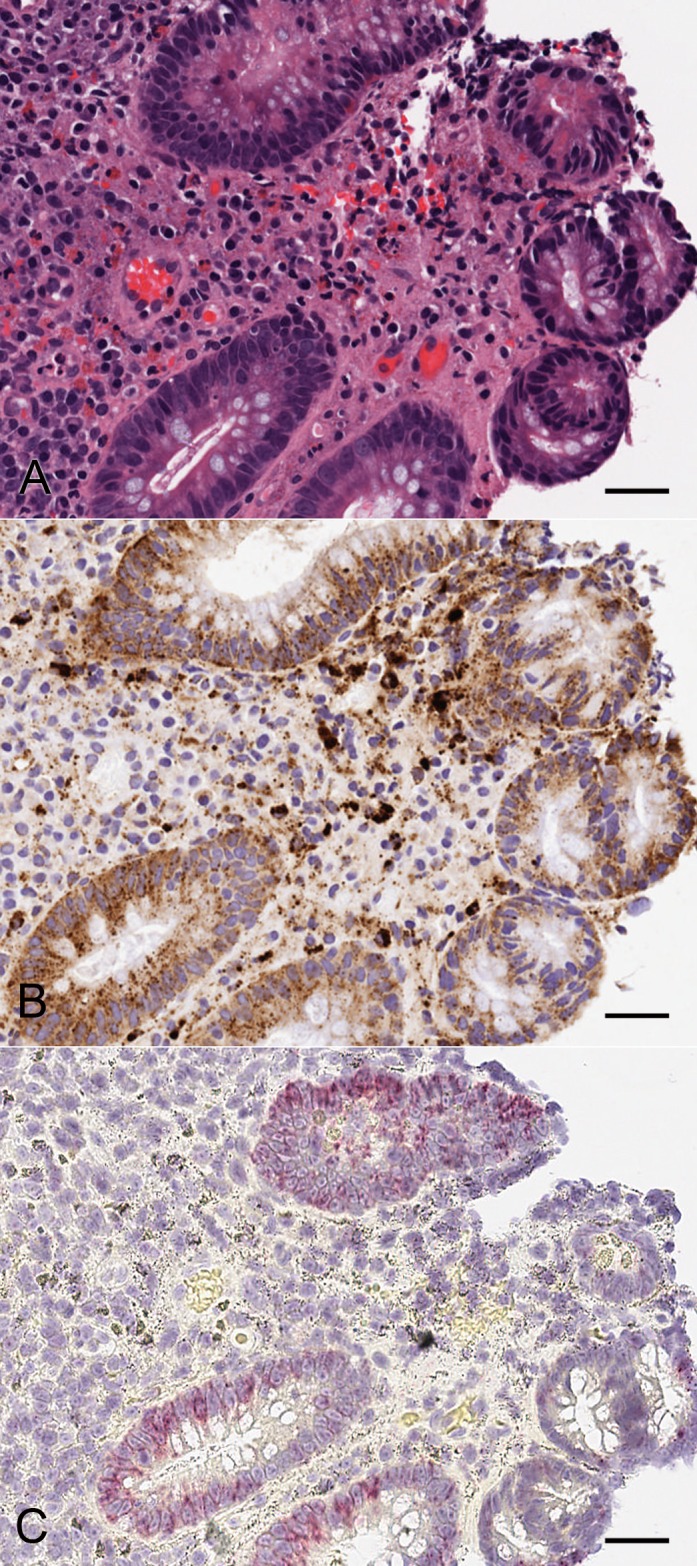
Comparative analysis of claudin-2 protein and mRNA localisation in adjacent sections from affected UC colon. (A) H&E staining, (B) IHC claudin-2 staining and (C) ISH claudin-2 staining. The localisation of claudin-2 protein (IHC staining) correlates to observed mRNA (ISH staining) pattern. The scale bars represent 50 μm.

Since the ISH staining in formalin fixed cells corresponded with the ICC on cells, we investigated mRNA expression of claudin-2 in FFPE human colon biopsy sections. In healthy colon samples and unaffected areas of colon from UC patients there was very low or no expression of claudin-2 mRNA (Fig 3C and 3D). Where claudin-2 mRNA expression was present, the signal was restricted to epithelial cells and was not present in cells in the lamina propria, including macrophages. In affected samples used for this initial method development work, mRNA expression was highest in crypts that showed significant pathology (crypt destruction) albeit less in even more severely diseased tissue which had lost crypt architecture through ulceration (Fig 3E and 3F). In addition, the samples tested with the sense probe (negative control) showed little or no signal in tissue (data not shown). Comparison of serial sections of human colon used for IHC with 12H2 and ISH with the claudin-2 probe, showed that the intensity of claudin-2 protein and mRNA signal was comparable within the epithelial cells of the crypts but claudin-2 mRNA expression was absent within the lamina propria (Fig 4). We therefore concluded that a claudin-2 mRNA ISH assay was more likely to be suitable for developing further as an assay with clinical utility.

**Fig 5:**
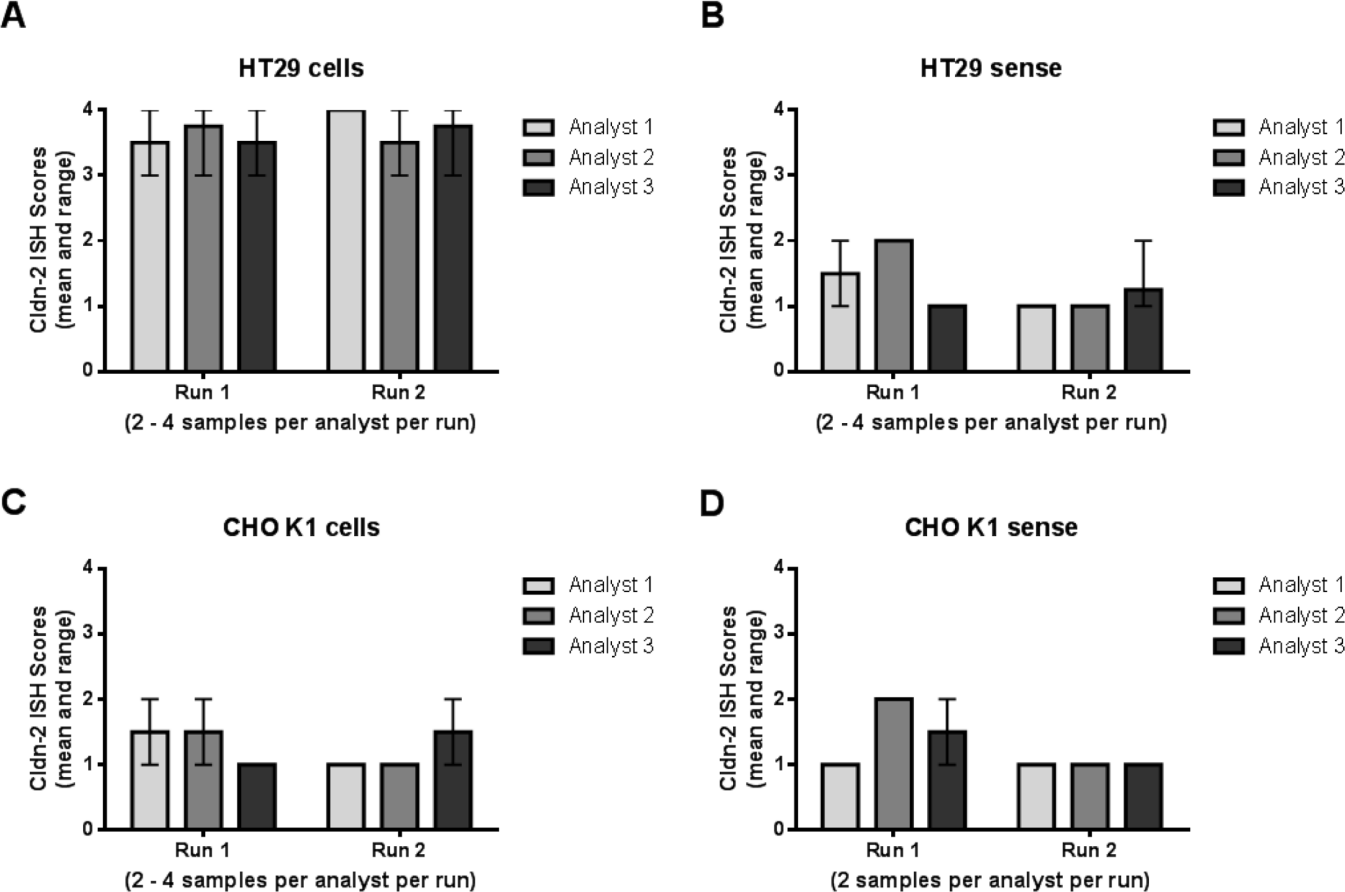
Assay variability for the Claudin-2 ISH method using FFPE cell controls. Three analysts each performed 2 independent analytical runs using positive (HT29) (A and B) and negative (CHO K1) (C and D) Formalin Fixed Paraffin Embedded (FFPE) cell lines. Cells were stained with claudin-2 (Cldn-2) probe (VA1-12003-01) (A and C) or Sense probe (VA1-14363-01) (B and D) from Panomics. Variability within & between analytical batches (performed by a single analyst) was minimal. Moreover, no analyst bias was observed across the 3 analysts’ performance. Data presented are ISH scores assigned by a single trained analyst.

**Fig 6:**
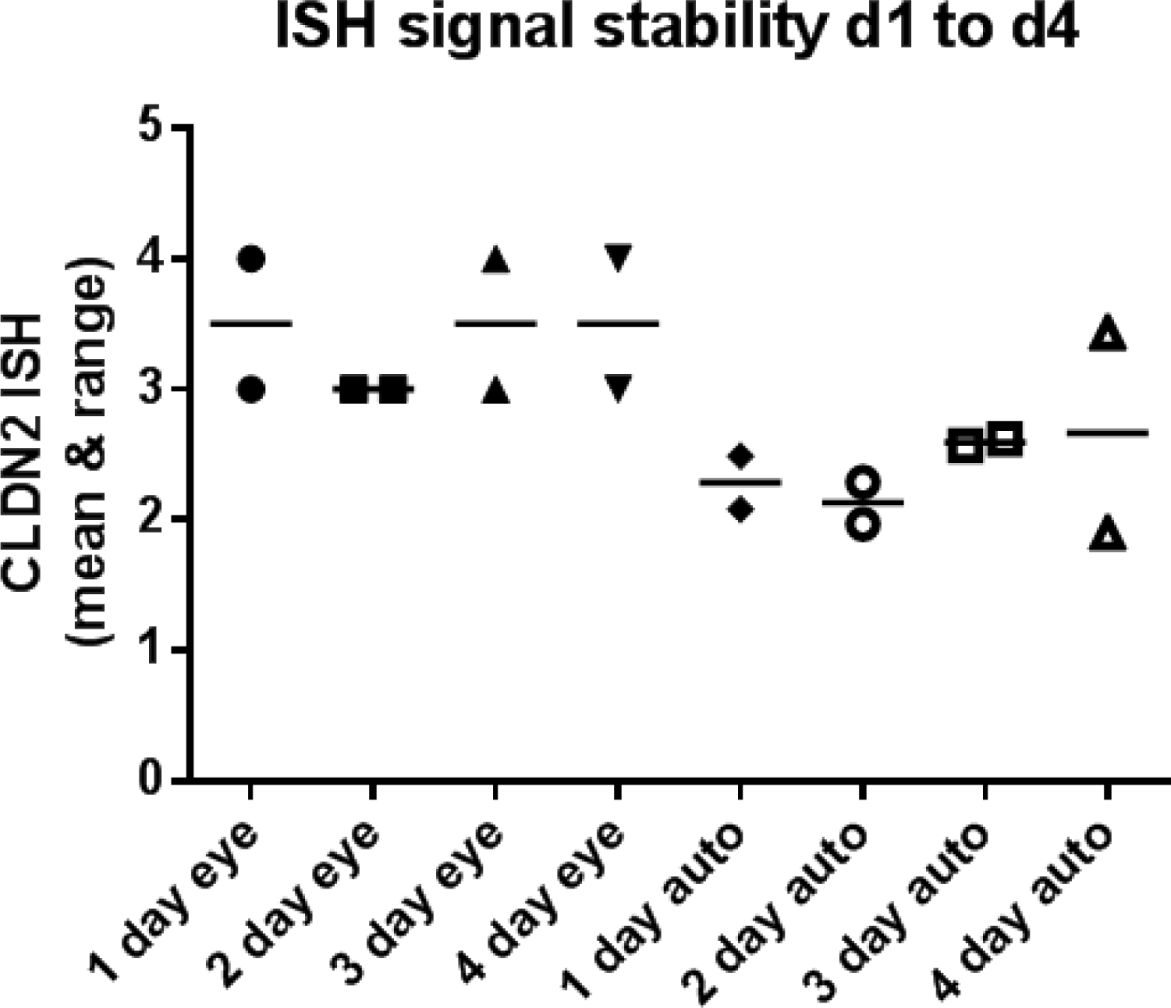
Effect of prolonged time intervals (> 1 day), following biopsy section pre-treatment, on ISH signal outcome. Samples were analysed in the same analytical run. (A) shows the variation in claudin-2 (Cldn-2) ISH signal by (eye) & by (auto) computer algorithm on sequential days (from 1 to 4) after slide pre-treatment step. Minimal variability is observed over the time period.

In order to validate the claudin-2 ISH assay for clinical regulatory bioanalytical deployment it was necessary to generate a quality control (QC) sample that would show a consistent level of claudin-2 mRNA signal. Tissue blocks contain a finite number of samples and, it was postulated within the team, would potentially have a higher likelihood of significant variation due to the focal nature of UC; therefore these were regarded as not being optimally suited for providing a consistent QC sample. As a result, HT29 cells endogenously expressing claudin-2 were used for all validation work and quality control. Negatively expressing CHO cells were used as a negative control. The accuracy and precision assessments within and between analytical runs, and between multiple analysts, were determined by analysis of sections of these positive and negative cell blocks stained either with the claudin-2 probe or a sense probe, which should not bind claudin-2 mRNA. Values were determined via by eye scoring (by a single blinded analyst) and also using automated image analysis (to avoid human scoring bias) of positive signal. Claudin-2 positive HT29 cells showed significantly higher claudin-2 ISH scores than the claudin-2 negative CHO cells and also positive or negative cells treated with the sense probe. Data showed that the performance of the assay was consistent within and between analytical runs, and that performance was not influenced by using multiple analysts (Fig 5A–5D). Moreover, no impairment of signal intensity was observed in “pre-treated” slides stored at room temperature, following dewaxing, for up to 4 days (Fig 6).

### Consistency of staining biopsies

**Fig 7:**
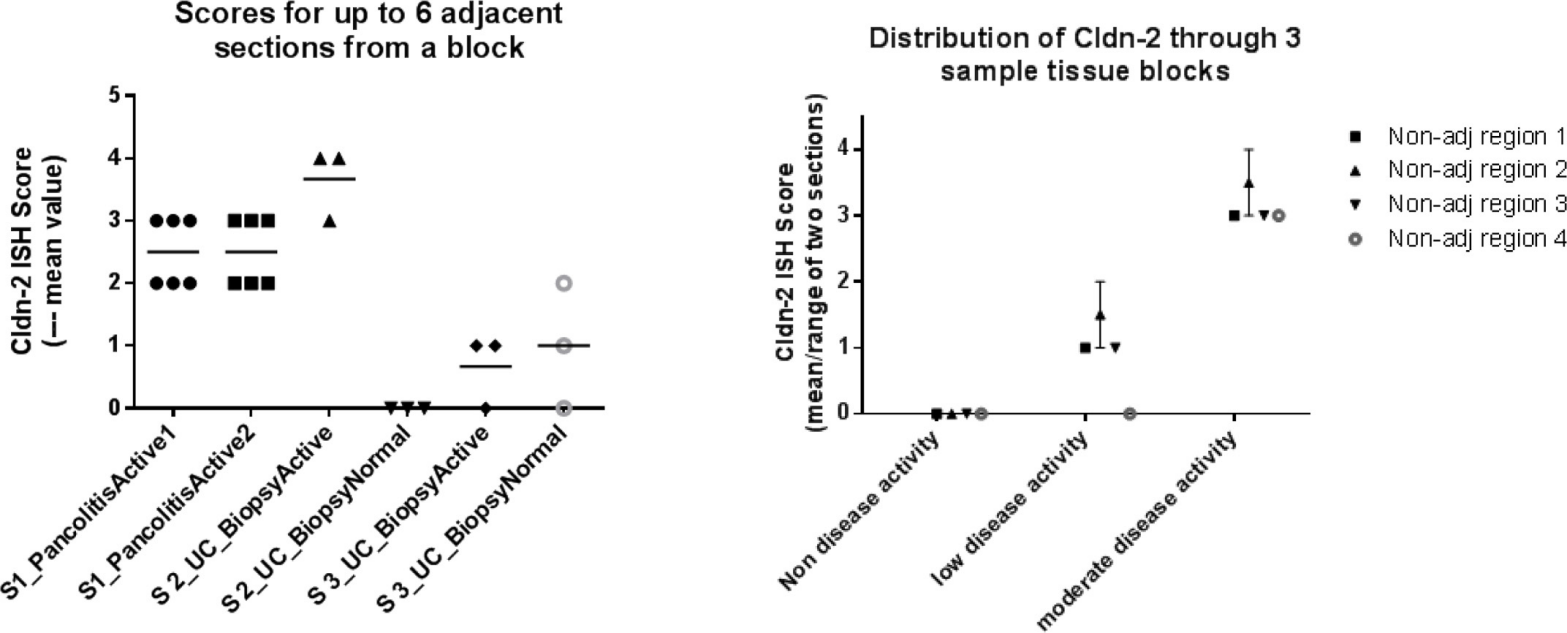
Investigating the potential variability of biomarker signal for adjacent and non-adjacent sequential biopsy sections. One micro-tomed 4 μM tissue section was generated (per datapoint) and ISH stained for claudin-2 mRNA distribution. Variability in the claudin-2 signal was investigated in (A) adjacent sections (3–6 novel sections per tissue) generated from a pancolitic (completely) active subject, and active & non-active UC material from two further subjects and in (B) the mean of two (adjacent) sections taken from four non-adjacent (≥ 24 μm distance apart).sites within a tissue biopsy, generated from 3 subjects who had non, low & moderate activity. In total, nine biopsies were analysed, and signal variability appears to be minimal within a specific biopsy.

The variation of claudin-2 distribution was evaluated in endoscopically affected and unaffected human UC biopsies from six individuals. Several sections were cut from an individual persons’ biopsy block; nine independent biopsies, at different grades of endoscopical disease activity, in total were evaluated. The variation of claudin-2 biomarker distribution in different levels of the biopsy was assessed in sections taken from 3–6 “adjacent” (0 μm distance apart) sections from the apical face of the biopsy only or 2 “adjacent” (4 μm distance apart) sections from four “non-adjacent” regions (approximately ≥ 24 μm distance apart) through the tissue block (including apical face). The differences between observed ISH scores from different sections of the biopsy, or between different samples from the same individual, were typically zero or one. As concluded from this assessment, the human UC tissue samples indicated comparable levels of claudin-2 expression between sections within a biopsy (Fig 7). These data provided confidence in the method to generate consistent results, despite the restricted the number of samples evaluated.

### Use of ISH as an additional tool for confirming target engagement

**Fig 8:**
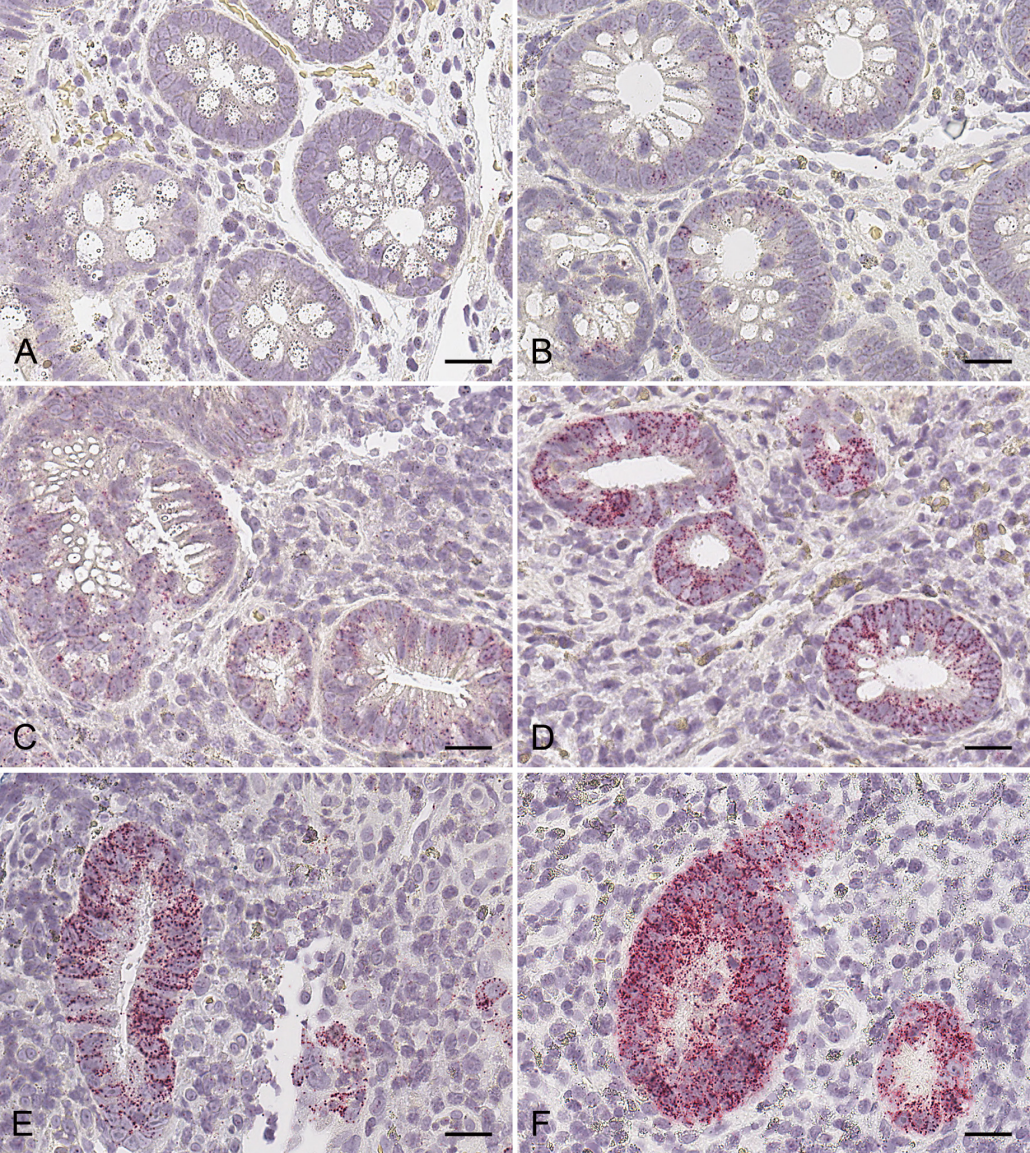
Representative grades of ISH claudin-2 staining in UC colon biopsies as disease severity increases. The level of disease severity was determined, by a trained pathologist, as (A) 0, none (B) 1, low, (C) 2, slight, (D) 3, moderate, (E) 4, marked and (F) 5, severe. The scale bars represent 50 μm.

The scoring system for claudin-2 mRNA expression established by the pathologist discriminated between the different levels of biomarker distribution and intensity. Fig 8 shows example biopsy sections of claudin-2 ISH staining that had been scored with a range of different grades (0–5) by the pathologist.

**Fig 9:**
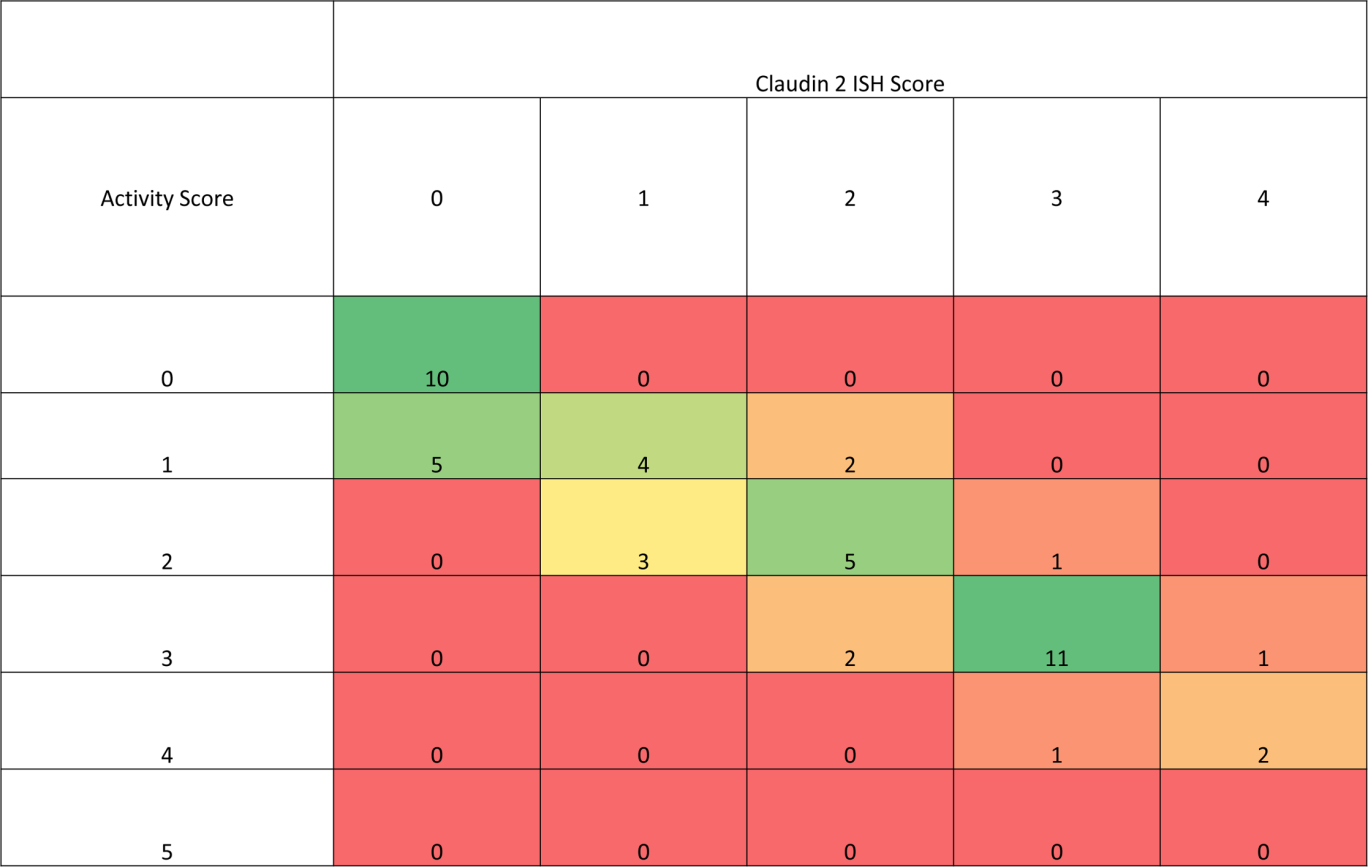
Heat-map (with sample frequency) showing the association between the Claudin-2 ISH score and a microscopical histopathological activity index. ISH scores and the activity index evaluations by the scoring pathologist were measured in the same biopsy sections from in 47 (affected and unaffected) UC biopsy sections used in the validation of the assay.

**Fig 10:**
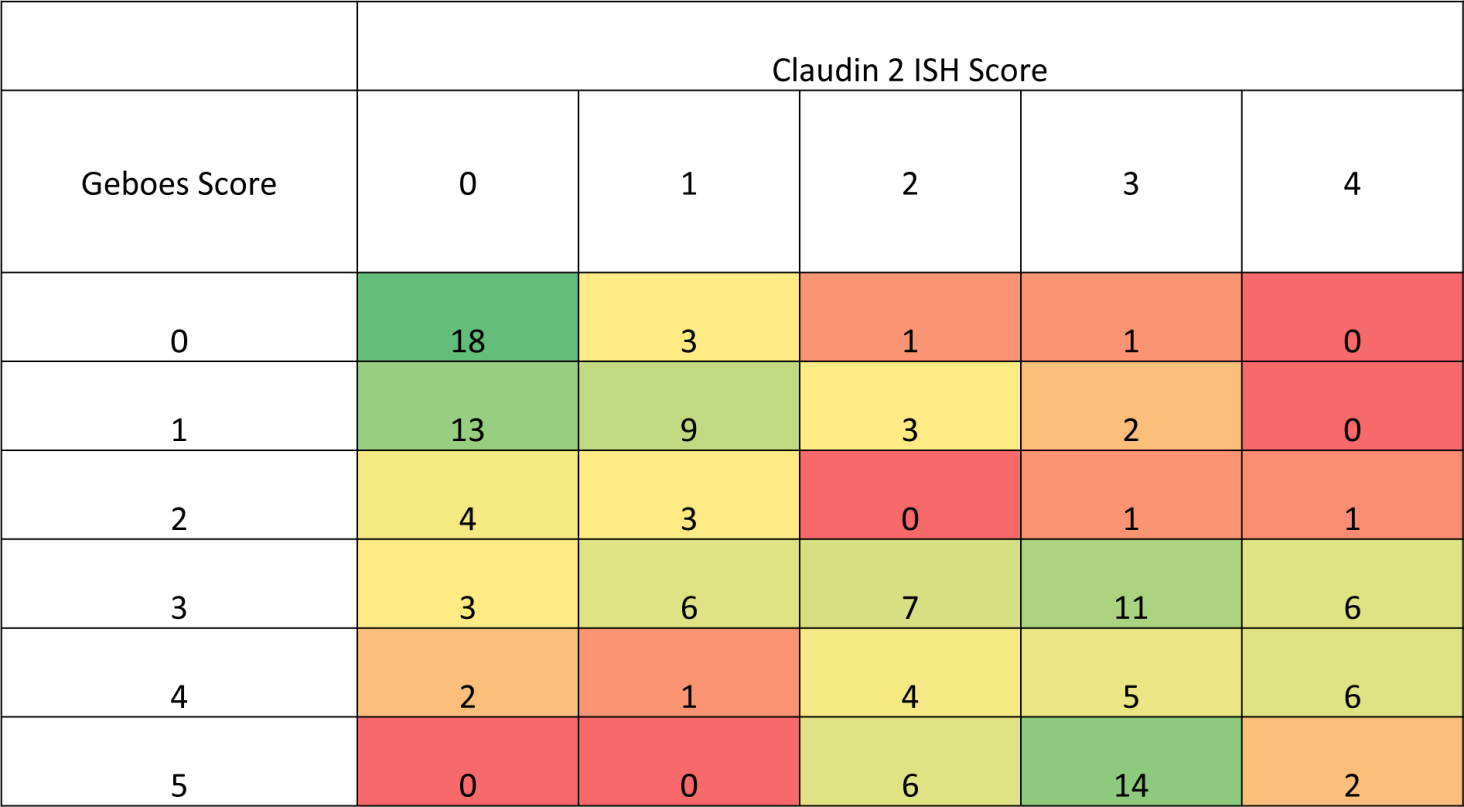
Heat-map (with sample frequency) showing the association between the Claudin-2 ISH score and the Geboes score. ISH and Geboes scores were measured in adjacent biopsy sections from in 132 (affected and unaffected) UC biopsies.

As part of the validation, the pathologist also gave an “activity” score (0–5) based on their interpretation of the microscopical histopathological feature of the tissue section. In the small population of biopsy samples (& sections) used within the validation (i.e. 47 in total), there appeared to be a trend of low ISH score with a low “activity index” score and vice versa (Fig 9). To determine if this was a real trend, the validated assay, including scoring system, was utilised on a larger cohort of biopsies (132 baseline samples) from a clinical study and showed that, in general, a higher microscopical Geboes score (indicating microscopically more severe disease), was associated with a high claudin-2 ISH score (higher claudin-2 mRNA expression; Fig 10). From a descriptive perspective, clinical study baseline samples with a low Geboes score (two or below) tended to have a low claudin-2 score (one or below) and those with a higher Geboes score (three or above) tended to have a higher claudin-2 score (two or above). It was notable that no samples with the highest Geboes score (five) had a claudin-2 score of less than two, or a score of five (the highest claudin-2 score), i.e. the claudin-2 ISH assay could not specifically discriminate samples with the highest Geboes score. From a statistical perspective, the Goodman and Kruskal Gamma value [21] for the table shown in Fig 10 is 0.65, with a 95% confidence interval of (0.56, 0.75). This measure takes the value +1 when the data are concentrated in the upper-left to lower-right diagonal for tables where both variables are ordered in the same direction. If both variables are independent the value is zero. The significant difference from zero provides evidence that the Geboes Scores and the ISH Claudin-2 scores are not independent. Since the value is towards +1 we have clear evidence of a positive correlation between the two scores. Probabilistically, the Gamma value is the difference in probability of “like” rather than “unlike” orders for the two scores when two samples are chosen at random.

## Discussion

The aim of this work was to establish and validate a method for detecting claudin-2 expression in routine FFPE human colon biopsies, which could be deployed for regulatory clinical bioanalysis and enable us to test the hypothesis that claudin-2 is a proof-of-mechanism biomarker for Ulcerative Colitis therapies. It is well documented that commercially sourced antibodies, are frequently not as specific as suppliers advertise[22],[24]. Therefore, we employed a systematic screening cascade of the five most frequently published anti-claudin-2 antibodies to determine their ability to specifically detect claudin-2 protein in FFPE cell lines and tissue sections of human colon by IHC. This resulted in only one antibody (12H2) showing acceptable specificity for claudin-2 by Western blotting, immunofluorescence and immunocytochemistry. Despite the confidence gained from the screening procedure and also the 12H2 antibody use in a number of peer reviewed publications [5], [11], [10], the expression pattern we observed in FFPE human colon sections was not consistent with the anticipated localization of claudin-2, based on biological function. 12H2 displayed strong immunostaining in epithelial cells, much of which was punctate staining within the cytoplasm; 12H2 also showed strongly positive cells within the lamina propria. This same pattern was observed in both control and in UC samples. Whilst some of the staining pattern in epithelial cells was consistent with anticipated (“chicken wire-like”) tight junction protein localization (notably in UC samples), the punctate cytoplasmic staining in the epithelial cells and the positive staining in lamina propria cells was considered non-specific.

Mass spectrometry confirmed the specificity of the 12H2 antibody for claudin-2 protein, suggesting that the additional unexpected IHC staining was introduced by the fixation or processing of the tissue samples. Indeed, it is well-known that tissue processing methods may influence the integrity of tissue morphology and biomarker signal [9]. A similar pattern of immunoreactivity has been shown in human colon samples in other studies [11], [10] using antibodies from the same supplier (presumed to be the same clone), although the authors did not comment on the distribution of immunoreactivity. Interestingly Prasad *et*. *al*. (2005) [5] also used a claudin-2 antibody from the same supplier, again presumed to be the same antibody, but their study demonstrated claudin-2 immunoreactivity only in tight junctions of UC or Crohn’s patients. However, the Prasad *et*. *al*. [5] study used acetone fixation followed by glycol methacrylate embedding and not formalin fixation and paraffin embedding. We therefore suggest that the unexpected additional staining with 12H2 in our study may be an artifact following formalin fixation. Consistent with this, similar punctate claudin-2 immunoreactivity has been observed in the cytoplasm of FFPE mouse bronchiolar epithelial cells [27] and human gall bladder epithelial cells [28]. Furthermore, immunohistochemistry on frozen human colon sections, including those from UC patients was not reported to show the additional staining we observed using FFPE material [29], [6].

The majority of clinical study samples (within AstraZeneca) are collected as formalin fixed samples for both practical reasons and to ensure the greatest preservation of morphology for the pathologists’ assessment. Therefore, we had to adapt our approach to be able to assess claudin-2 (on internally generated samples), and so pursued the use of an mRNA ISH assay as an alternative target engagement biomarker assay. The ISH signal intensity for claudin-2 was found to correlate well with the IHC signal intensity specifically in the affected crypt epithelial cells, further supporting that the 12H2 antibody was detecting at least in part claudin-2 protein correctly. In contrast to IHC, ISH gave a distinct signal above background, suitable for manual scoring by a pathologist or potentially using automated image analysis.

A simple scoring algorithm was established for the ISH assay read-out, and subsequently formally assessed in line with the presently available 2011 FDA guidance (albeit draft) [30] on validation approaches for tissue staining methodologies. A tiered approach [31] was applied to perform the tests required to demonstrate the assay as “fit for purpose” [32] *e*.*g*. reproducibility within and between assays, between analysts, and environmental stability of analyte. The validation approach was partially driven by the challenges associated with obtaining high quality material and the unknown conditions influencing consistency of the biomarker signal throughout the biopsy material. For clinical deployment of this type of assay, it was important to have confidence in the robustness of the data generated, including low variability of the biomarker signal observed within same biopsy, as determined by our claudin-2 read-out. Our data indicated that the variability between or within analytical runs or between analysts was low, giving confidence to the consistency of data generated in large studies performed over time. Moreover, the active and inactive biopsies (as determined by the pathologist) showed low variability in claudin-2 signal in the multiple sections taken for analysis. This indicated that a single section from each biopsy (regardless of level within biopsy) could be interrogated and assumed to be representative of the biomarker expression throughout the biopsy sample.

Due to the low variability observed, only one tissue section per subject and location was used for analysis (rather than a mean of several analyzed tissue sections, as observed in some cancer biomarker assessments [33]) and thereby paralleling the clinically well-established regime for sampling and assessing microscopical activity (e.g. Geboes score). Hence this reduces workload and cost, and has the benefit of a clinical assay that adds value to the microscopical disease score evaluations derived from the same samples. To control for assay performance between experimental runs, sections from FFPE cell blocks were used as a positive control for staining. Although it technically would have been feasible to use human biopsy sections, this wasn’t realistically considered to be an economical or ethical option as samples would be difficult to obtain (as we had experienced during method development & validation) and there was a good alternative available. Also, the limited number of sections that can be obtained from a single biopsy would not be compatible with standardization across any greater number of samples.

The simpler composition of the cell block sections combined with the need to only run one positive control for a batch of samples allowed automated quantification of the claudin-2 mRNA signal in these samples. Due to the inherent complexity of the microscopical characteristics for activity in diseased mucosa (e.g. epithelial and crypt derangement), attempts to perform automated analysis of the ISH signal were not considered to be successful. Evaluating parameters such as stability of the ISH signal over time (up to 4 days) following dewaxing in “pre-treated” slides, supported analysis of a larger number of samples at each timepoint, hence a more practical and flexible approach without compromising quality.

Once validated as above, and having observed a potential trend between activity and ISH score on small number of validation samples, we deployed the ISH assay on baseline clinical samples from a mild-severe UC patient population. Using these samples, it was possible to establish that, in general, the microscopic Geboes score obtained from the same sample correlated well with the claudin-2 biomarker score. However, with the highest Geboes score (5) there were no correlating samples that showed a claudin-2 score of 5. Indeed most of these samples with a Geboes score of 5, showed a claudin-2 score of 3. This discordance is likely due to the fact that with the Geboes score of 5, features of ulceration and crypt degeneration also result in overt loss of epithelial cells and consequently loss of the structures where expression of the claudin-2 signal would be expected. It is therefore anticipated that the claudin-2 signal would be lower at higher Geboes scores where the epithelial/crypt cells have been lost. This is the first study to relate the microscopical severity of disease to claudin-2 expression and our results are consistent with others showing an overall increase in claudin-2 in UC samples [2], [5]. Accordingly, this work supports the use of claudin-2 as a useful biomarker representing the pathogenesis of UC.

## Conclusions

This study has highlighted that, even with antibodies used in peer-reviewed publications, there is a need for careful validation of antibodies for specific applications, particularly IHC where protein epitopes may become modified by the fixation process. Moreover we have demonstrated a novel validation strategy for developing a fit-for-purpose ISH assay that can be clinically deployed within drug projects targeting UC, known to have changes in claudin-2 expression. This strategy can be applied to future studies which require the development and validation of tissue based biomarker assays for clinical deployment. The claudin-2 ISH method described here thus offers the potential to be used in support of established methods, and may be a more sensitive approach to confirm barrier function/mucosal healing in mild/moderate active UC patients. Increasing Claudin-2 activity was seen to correlate very well with our morphological activity score where this reflects increasing grades of crypt epithelial inflammation and epithelial degeneration/disintegration. Understandably, in the most severe grades with frank ulceration where the crypt epithelial structures are lost all together, the Claudin-2 activity was also seen declining or absent. As a consequence, Claudin-2 based scoring can be expected to correlate well with activity-related components of commonly used microscopical scores like Riley or Geboes, but for the same reason also here can be expected to dissociate with severe crypt loss/ulceration grades. Further studies, on larger sample numbers, are needed to confirm the validity for assessment of disease activity and relapse-prediction in the general UC patient population.

## Supporting Information

S1 Fig Immunofluorescence antibody screen data for lead candidate anti-claudin-2 antibody (32–5600). Figure shows direct immunofluorescence staining of GFP-CLDN2 overexpressing CHO cells stained with (a) the 32–5600 (12H2) antibody against Claudin-2 (red), counterstained with DAPI, (B) GFP fluorescence (green) and (C) combined image showing co-localisation of antibody staining with GFP label. Panel (D) shows endogenously expressed CLDN2 in HT29 cells (using 32–5600 (12H2) antibody).
(TIF)

S2 Fig Mass spectrometry antibody screen data for lead candidate anti-claudin-2 antibody (32–5600). (A) gel separation of 35-5600-immunoprecipitated T84 cell extracts. (B) Specific bands were characterised by mass spectrometry.
(TIF)

S3 Fig Lysozyme tissue staining antibody screen data for lead candidate anti-claudin-2 antibody (32–5600). Assessing cross reactivity of antibody staining in human tissue. Tissue was incubated with (A) 32–5600 only, (B) 32–5600 plus lysozyme blocking protein, (C) an anti-lysozyme antibody only, (D) with an anti-lysozyme antibody plus lysozyme blocking protein. (A) shows staining pattern of Claudin-2; punctate staining in the epithelium is present as well as the intense staining of macrophage like cells in the lamina propria. (B) shows identical staining pattern to Claudin-2 alone. (C) strong signal seen in macrophage like cells within the lamina propria. Note: no punctate staining seen in epithelial cells. (D) shows that the lysozyme signal essentially disappears. From the pattern of distribution we can say that the Claudin-2 antibody is not picking up lysosome proteins and this is supported by the peptide block for lysozyme where a decrease in claudin-2 signal is not seen.
(TIF)

S4 Fig Lysozyme western blot antibody screen data for lead candidate anti-claudin-2 antibody (32–5600). Figure shows (A) results and (B) experimental design for lysozyme peptide blocking experiment.
(TIF)

S5 Fig Western blot antibody screen data for rejected candidate anti-claudin-2 antibody (IMG80487). Western blot of cell lysates and recombinant CLDN2-GST protein was probed with IMG80487 anti-CLDN2 antibody. Antibody recognised recombinant protein. A band of >20 kDa was seen in endogenously expressing HT29 & T84 cells. Some additional faint non-specific bands were also detected around 50–60 kDa. Negative control CHO-K1 cells did show some non-specific staining at >100 KDa, although specific staining of overexpressing CLDN2-GFP protein was present. This antibody may be fit for purpose if titrated out and validated in final assay.
(TIF)

S6 Fig Immunofluorescence antibody screen data for rejected candidate anti-claudin-2 antibody (IMG80487). CLDN2-GFP & labelled IMG80487 images overlaid, showing IMG80487 is compatible for detecting CLDN-2 in immunofluorescence against overexpressing CHO-K1 cells. Staining of endogenously expressing CLDN2 HT29 cells however, was unsuccessful. Amplification of the signal may resolve this, further work would be required.
(TIF)

S7 Fig Selection of antibody screening data for rejected anti-claudin-2 antibody (NBP1-67516). (A) Immunofluorescence data showing CHO-K1 overexpressing CLDN2-GFP, labelled with NBP1-67516. NBP1-67516 is compatible for detecting CLDN-2 in IF (protocol needs optimising). (B) Western blot of cell lysates and recombinant CLDN2-GST protein was probed with NBP1-67516 anti-CLDN2 antibody. Antibody recognised recombinant protein. A single band of >20kDa was seen in endogenously expressing HT29 & T84 cells. No staining was seen in negative control CHO-K1 cells, although overexpression of GFP-CLDN2 protein in CHO-K1 cells, also did not show expected staining.
(TIF)

S8 Fig Selection of antibody screening data for rejected anti-claudin-2 antibody (AP23596PU-N). (A) Immunofluorescence data showing CHO-K1 overexpressing GFP-CLDN2 protein, labelled with AP23596PU-N. Immunofluorescence data did not show specific binding. (B) Western blot of cell lysates and recombinant CLDN2-GST protein was probed with Acris Antibodies AP2359 anti-CLDN2 antibody. Antibody faintly recognised recombinant protein, which is most likely due to non-specific binding. A ladder of non-specific bands in endogenously expressing HT29,T84 cells was seen. AP2359 did not distinguish between non-transfected CHO-K1 cells and CHO-K1 cells overexpressing GFP-CLDN2 protein.
(TIF)

S9 Fig Western blot antibody screening data for rejected anti-claudin-2 antibody (51–6100). Western blot of equally loaded cell lysates and recombinant CLDN2-GST protein was probed with Invitrogen 51–6100 anti-CLDN2 antibody. Antibody failed to recognise recombinant protein, and picked up a ladder of non-specific bands in endogenously expressing HT29,T84. 51–6100 did not distinguish between non-transfected CHO-K1 and CHO-K1 cells overexpressing CLDN2-GFP protein.
(TIF)
