# Supplementary figures and images for: Claudin-2 Expression Levels in Ulcerative Colitis: Development and Validation of an In-Situ Hybridisation Assay for Therapeutic Studies
Source: PLoS One. 2016 Sep 6;11(9):e0162076. doi: 10.1371/journal.pone.0162076 (PMC5012586; doi:10.1371/journal.pone.0162076)

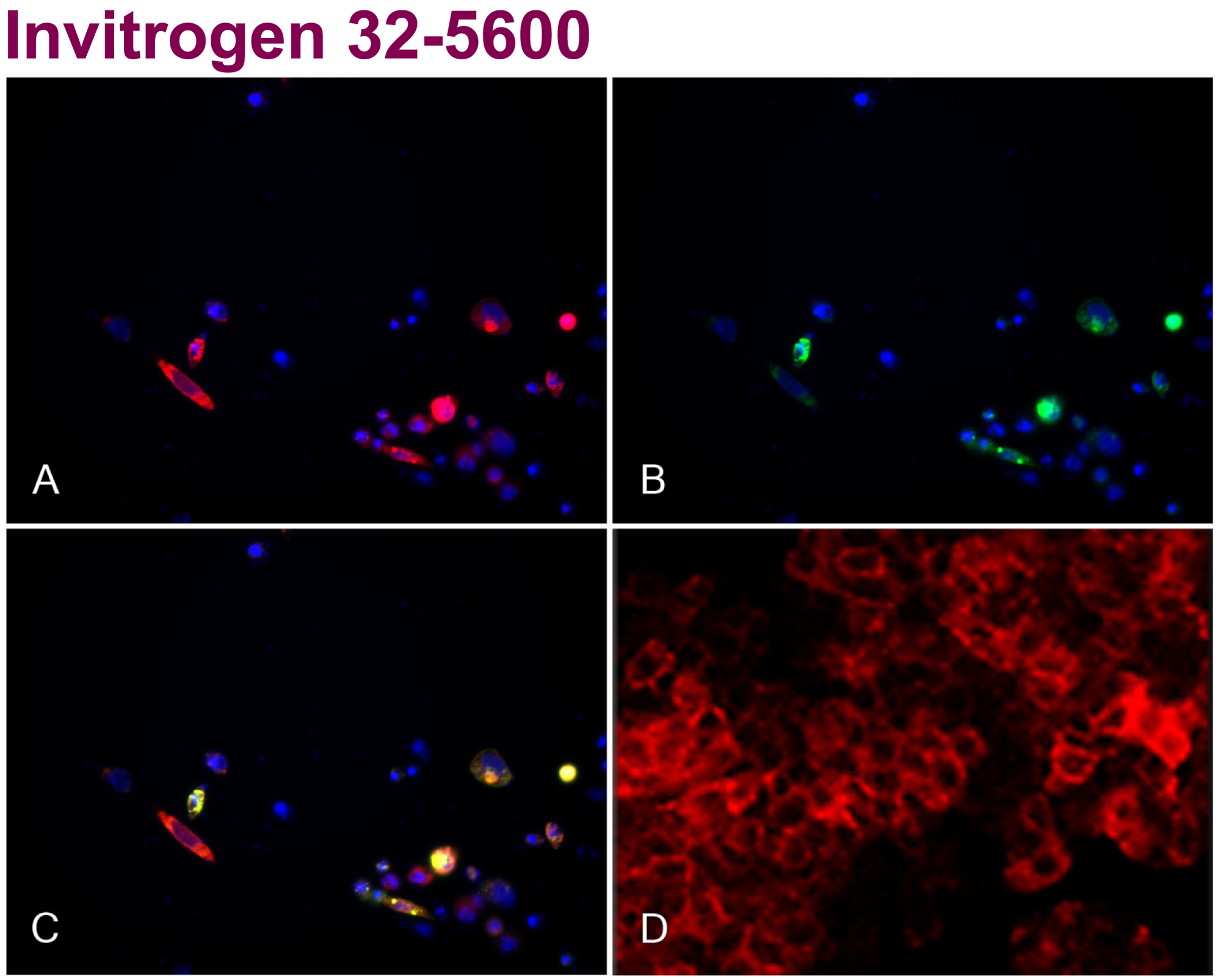

Supplement: S1 Fig — Figure shows direct immunofluorescence staining of GFP-CLDN2 overexpressing CHO cells stained with (a) the 32–5600 (12H2) antibody against Claudin-2 (red), counterstained with DAPI, (B) GFP fluorescence (green) and (C) combined image showing co-localisation of antibody staining with GFP label. Panel (D) shows endogenously expressed CLDN2 in HT29 cells (using 32–5600 (12H2) antibody). (TIF) [file pone.0162076.s001.tif]

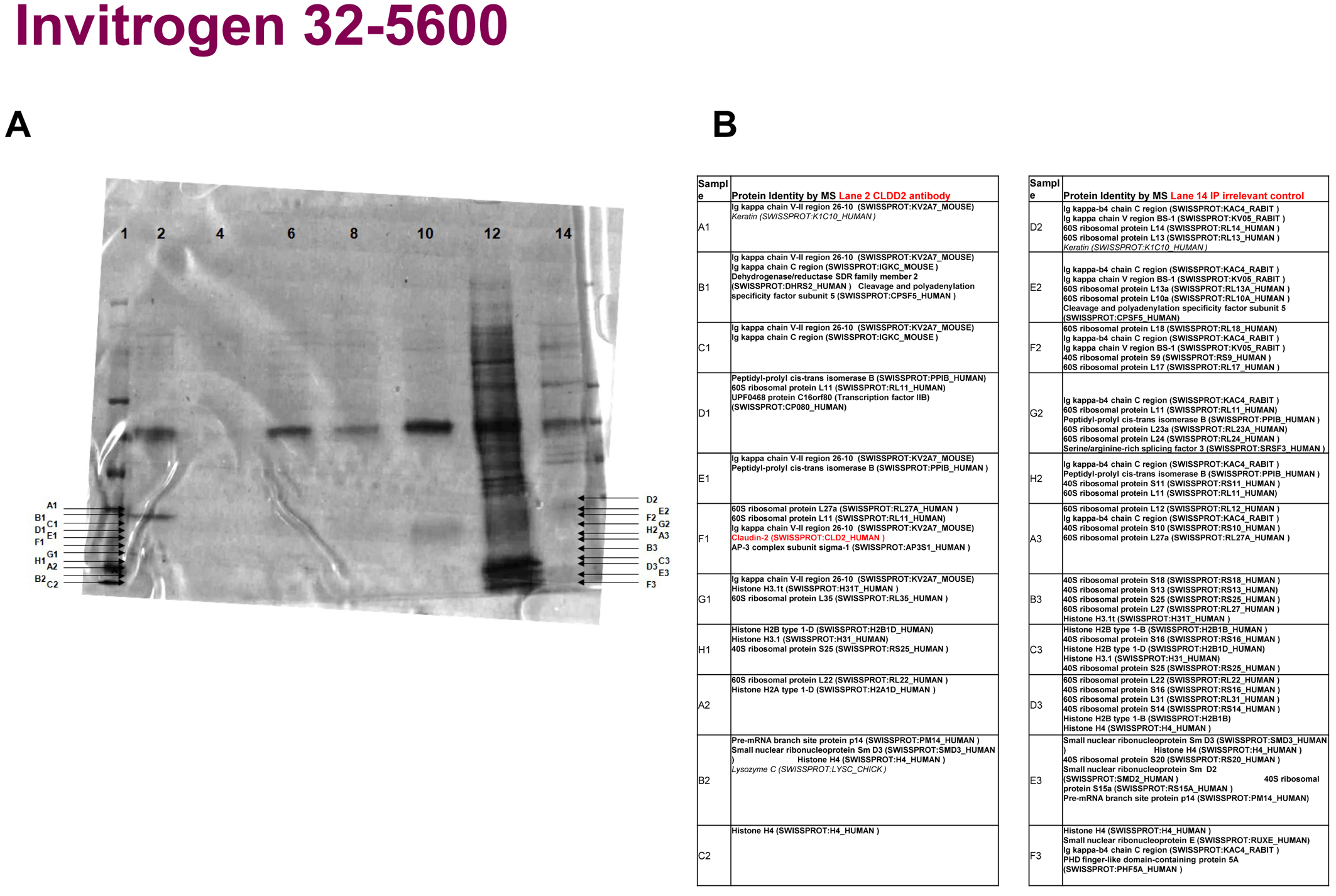

Supplement: S2 Fig — (A) gel separation of 35-5600-immunoprecipitated T84 cell extracts. (B) Specific bands were characterised by mass spectrometry. (TIF) [file pone.0162076.s002.tif]

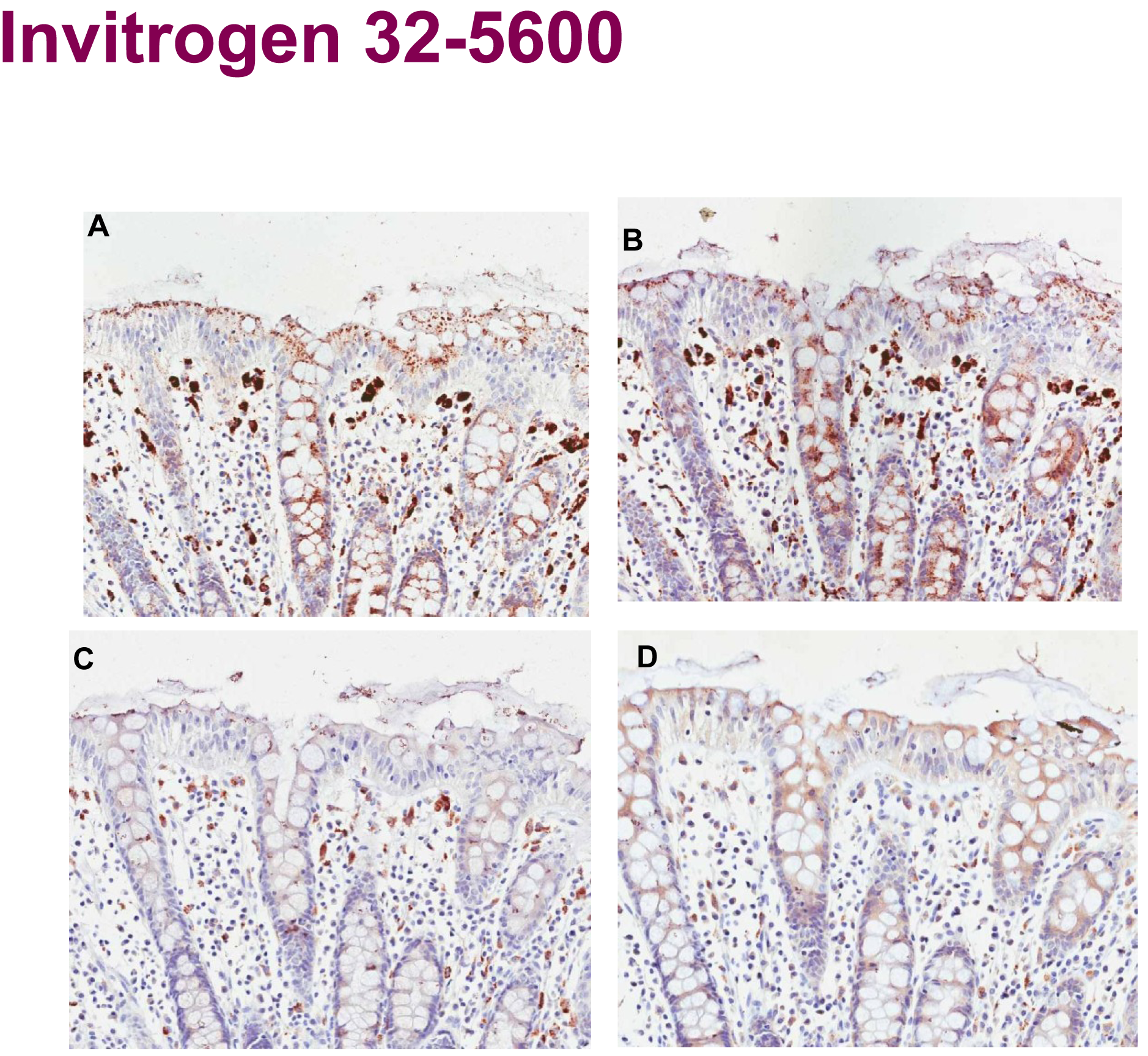

Supplement: S3 Fig — Assessing cross reactivity of antibody staining in human tissue. Tissue was incubated with (A) 32–5600 only, (B) 32–5600 plus lysozyme blocking protein, (C) an anti-lysozyme antibody only, (D) with an anti-lysozyme antibody plus lysozyme blocking protein. (A) shows staining pattern of Claudin-2; punctate staining in the epithelium is present as well as the intense staining of macrophage like cells in the lamina propria. (B) shows identical staining pattern to Claudin-2 alone. (C) strong signal seen in macrophage like cells within the lamina propria. Note: no punctate staining seen in epithelial cells. (D) shows that the lysozyme signal essentially disappears. From the pattern of distribution we can say that the Claudin-2 antibody is not picking up lysosome proteins and this is supported by the peptide block for lysozyme where a decrease in claudin-2 signal is not seen. (TIF) [file pone.0162076.s003.tif]

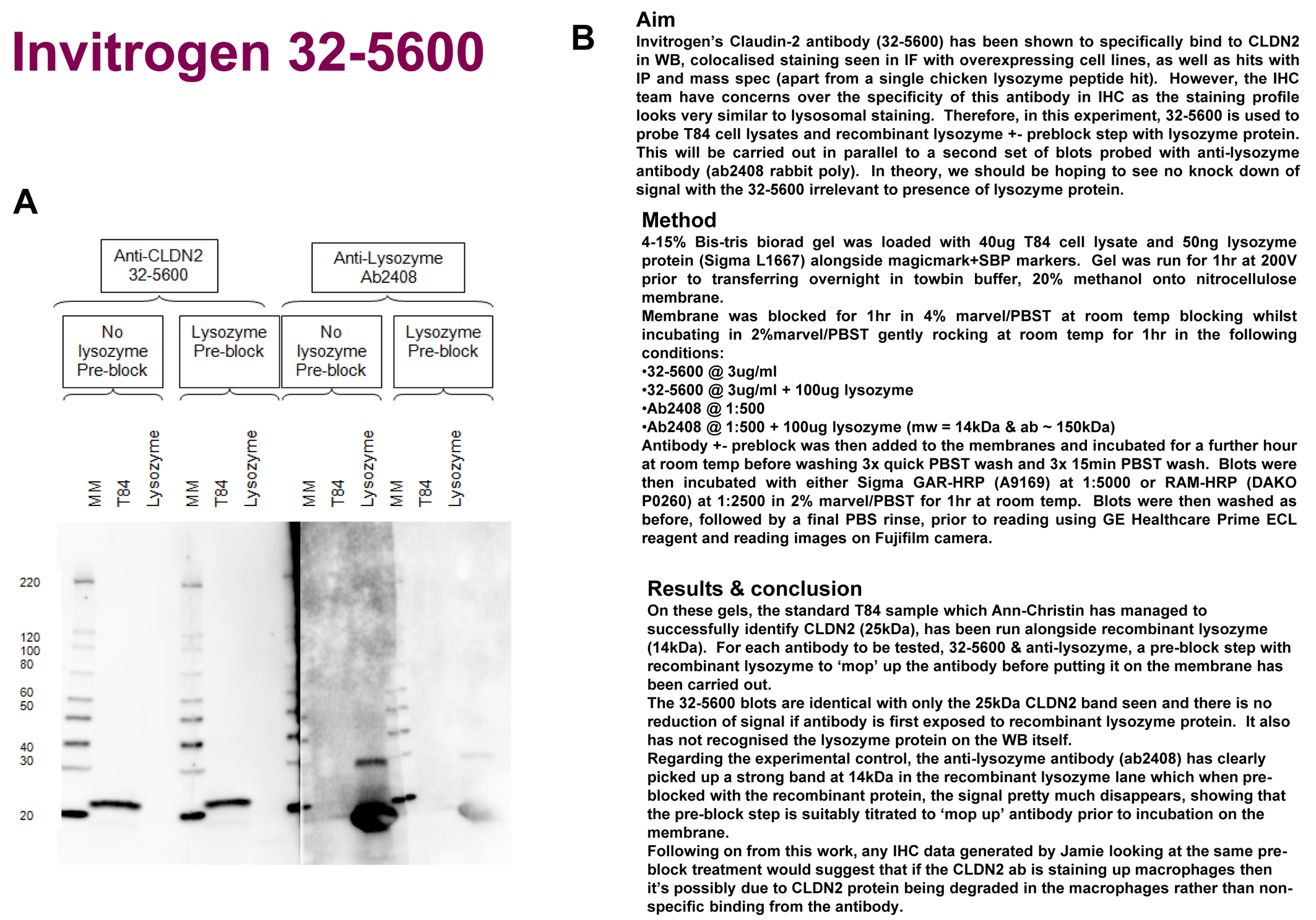

Supplement: S4 Fig — Figure shows (A) results and (B) experimental design for lysozyme peptide blocking experiment. (TIF) [file pone.0162076.s004.tif]

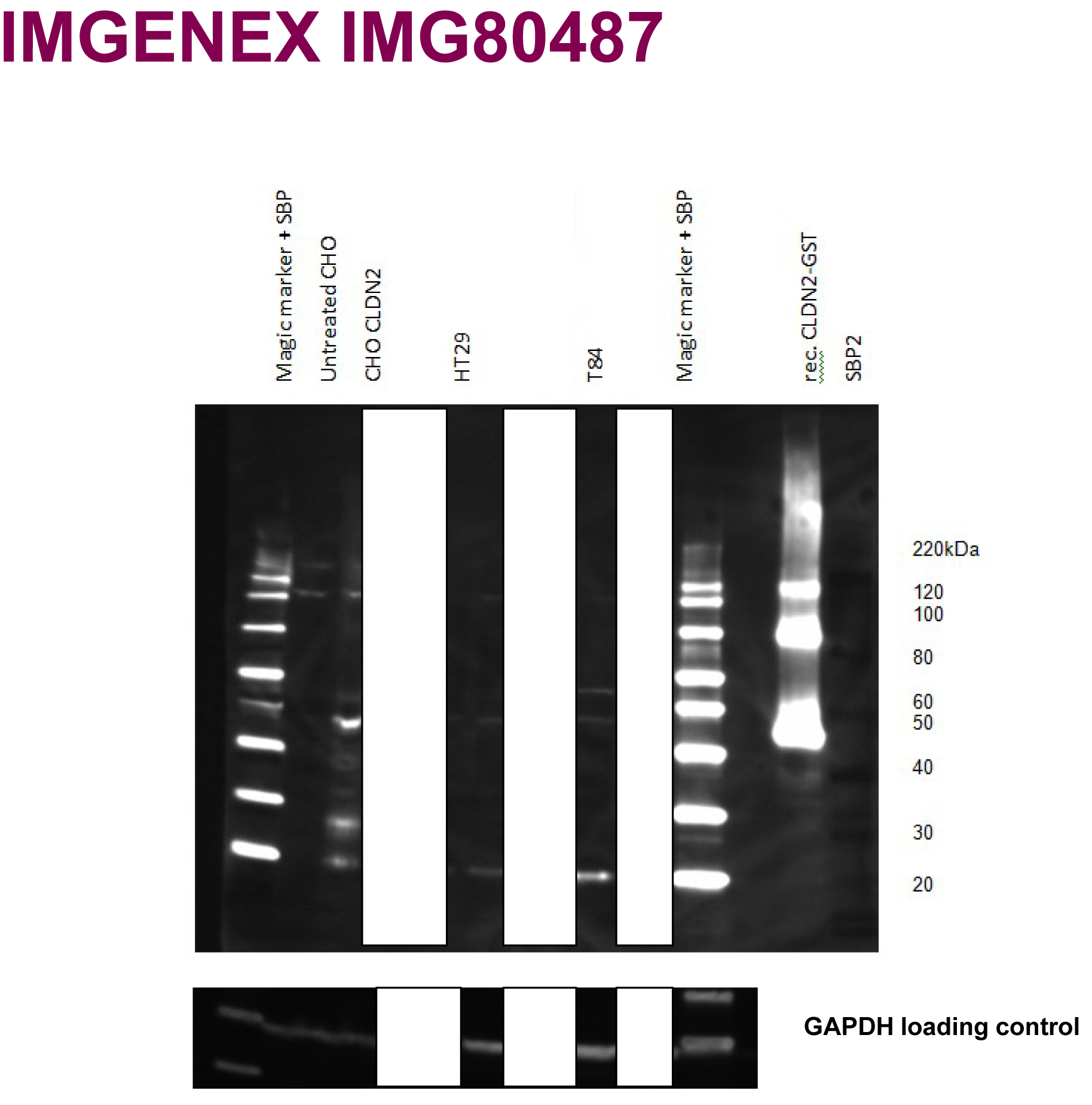

Supplement: S5 Fig — Western blot of cell lysates and recombinant CLDN2-GST protein was probed with IMG80487 anti-CLDN2 antibody. Antibody recognised recombinant protein. A band of >20 kDa was seen in endogenously expressing HT29 & T84 cells. Some additional faint non-specific bands were also detected around 50–60 kDa. Negative control CHO-K1 cells did show some non-specific staining at >100 KDa, although specific staining of overexpressing CLDN2-GFP protein was present. This antibody may be fit for purpose if titrated out and validated in final assay. (TIF) [file pone.0162076.s005.tif]

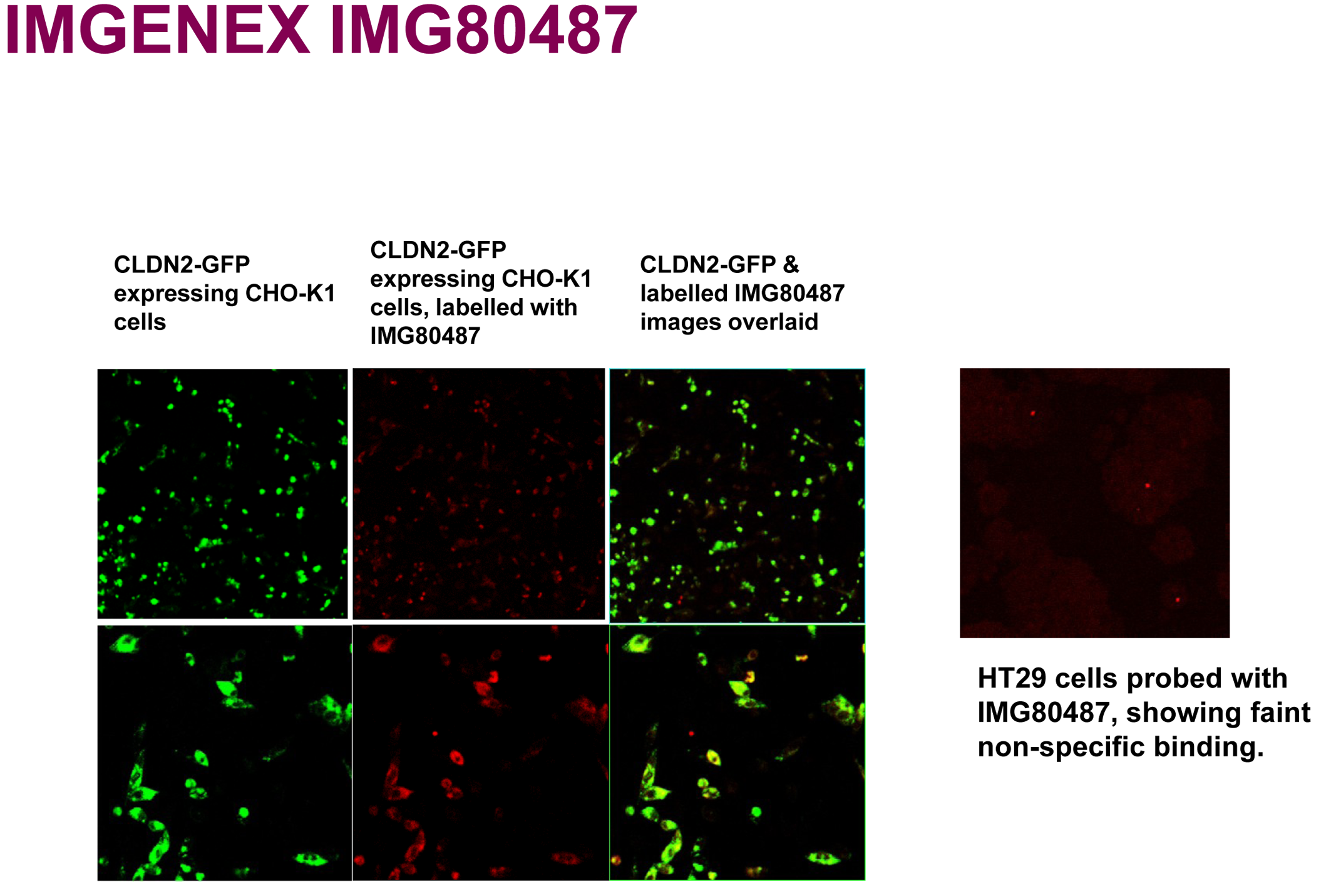

Supplement: S6 Fig — CLDN2-GFP & labelled IMG80487 images overlaid, showing IMG80487 is compatible for detecting CLDN-2 in immunofluorescence against overexpressing CHO-K1 cells. Staining of endogenously expressing CLDN2 HT29 cells however, was unsuccessful. Amplification of the signal may resolve this, further work would be required. (TIF) [file pone.0162076.s006.tif]

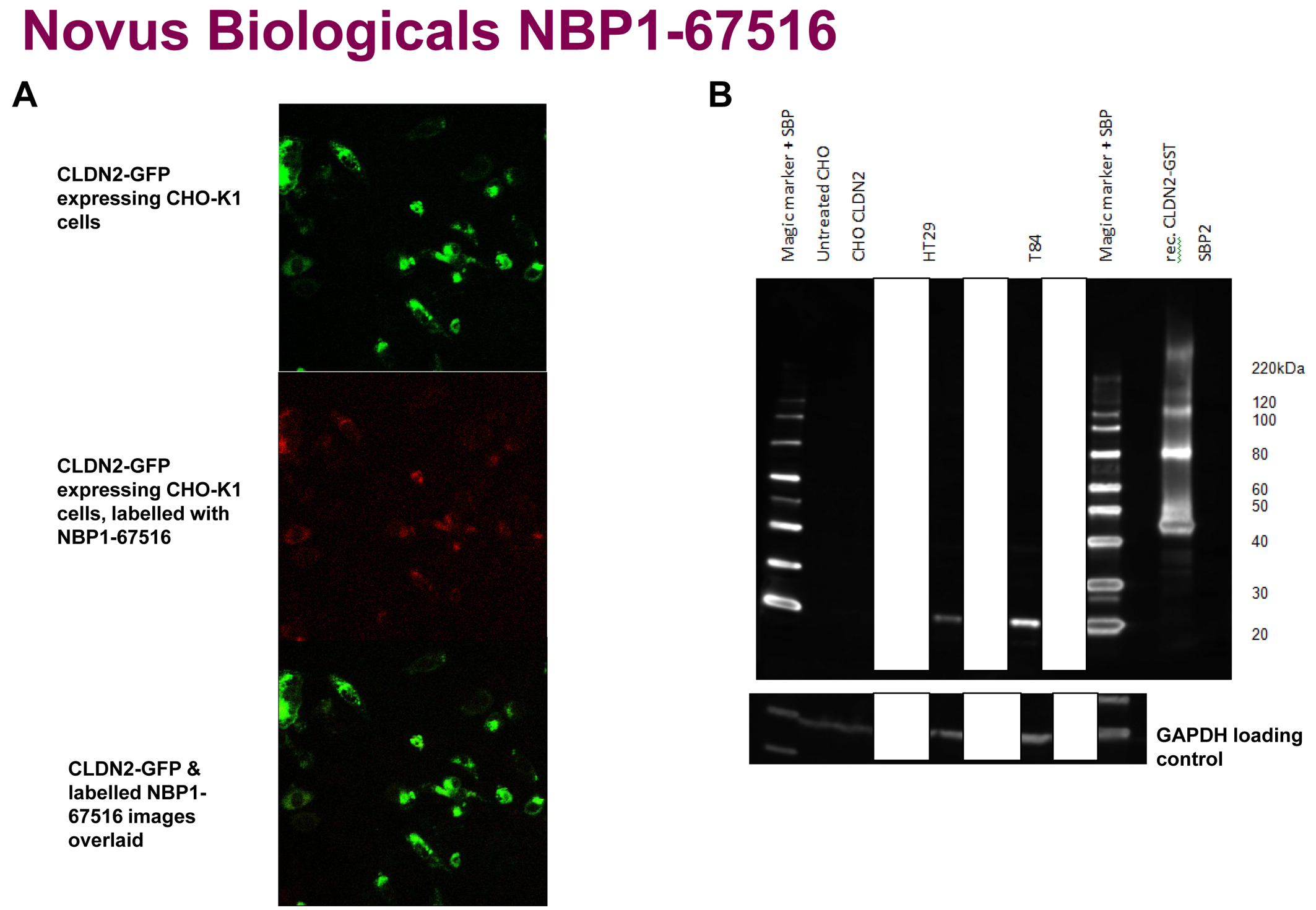

Supplement: S7 Fig — (A) Immunofluorescence data showing CHO-K1 overexpressing CLDN2-GFP, labelled with NBP1-67516. NBP1-67516 is compatible for detecting CLDN-2 in IF (protocol needs optimising). (B) Western blot of cell lysates and recombinant CLDN2-GST protein was probed with NBP1-67516 anti-CLDN2 antibody. Antibody recognised recombinant protein. A single band of >20kDa was seen in endogenously expressing HT29 & T84 cells. No staining was seen in negative control CHO-K1 cells, although overexpression of GFP-CLDN2 protein in CHO-K1 cells, also did not show expected staining. (TIF) [file pone.0162076.s007.tif]

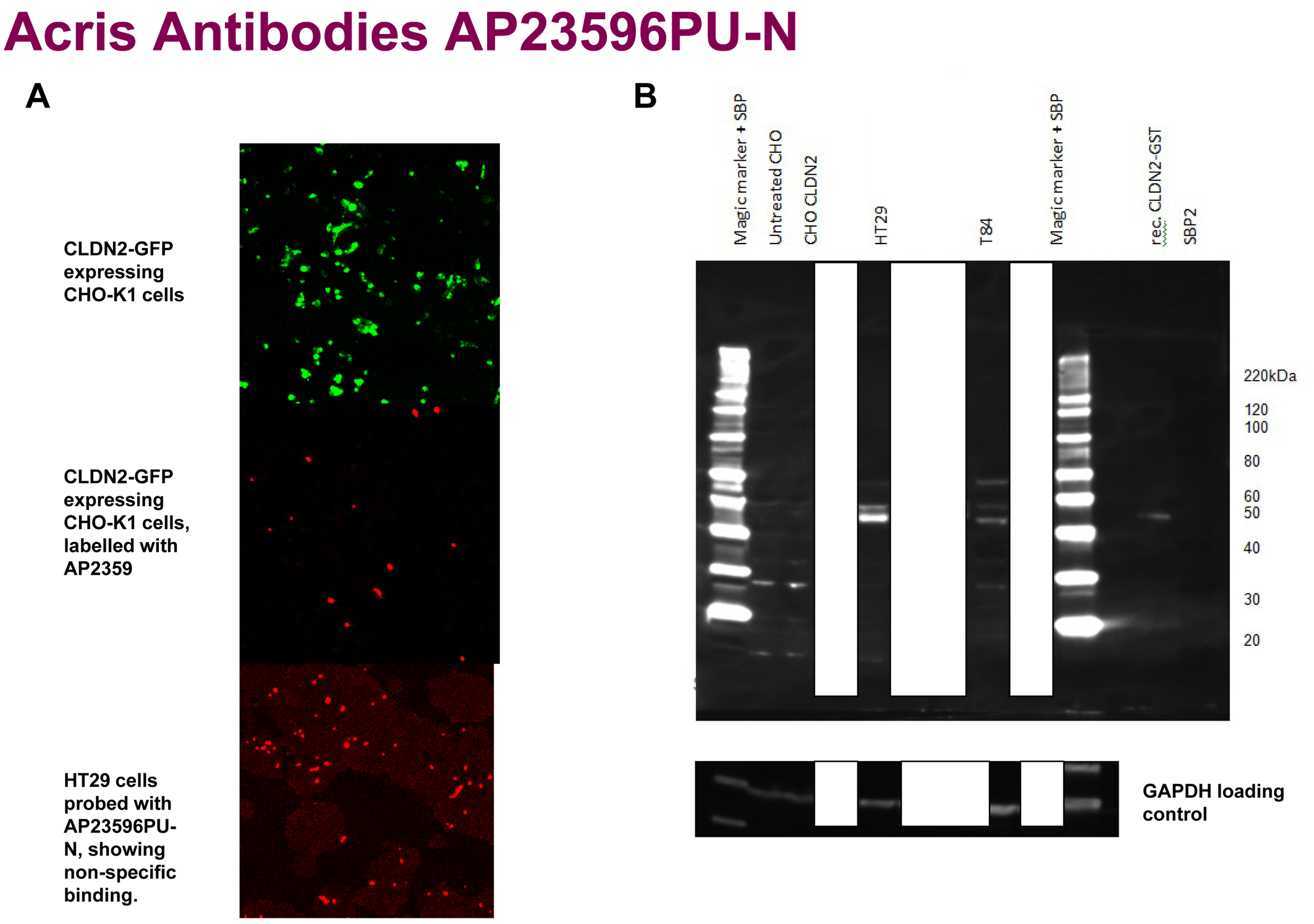

Supplement: S8 Fig — (A) Immunofluorescence data showing CHO-K1 overexpressing GFP-CLDN2 protein, labelled with AP23596PU-N. Immunofluorescence data did not show specific binding. (B) Western blot of cell lysates and recombinant CLDN2-GST protein was probed with Acris Antibodies AP2359 anti-CLDN2 antibody. Antibody faintly recognised recombinant protein, which is most likely due to non-specific binding. A ladder of non-specific bands in endogenously expressing HT29,T84 cells was seen. AP2359 did not distinguish between non-transfected CHO-K1 cells and CHO-K1 cells overexpressing GFP-CLDN2 protein. (TIF) [file pone.0162076.s008.tif]

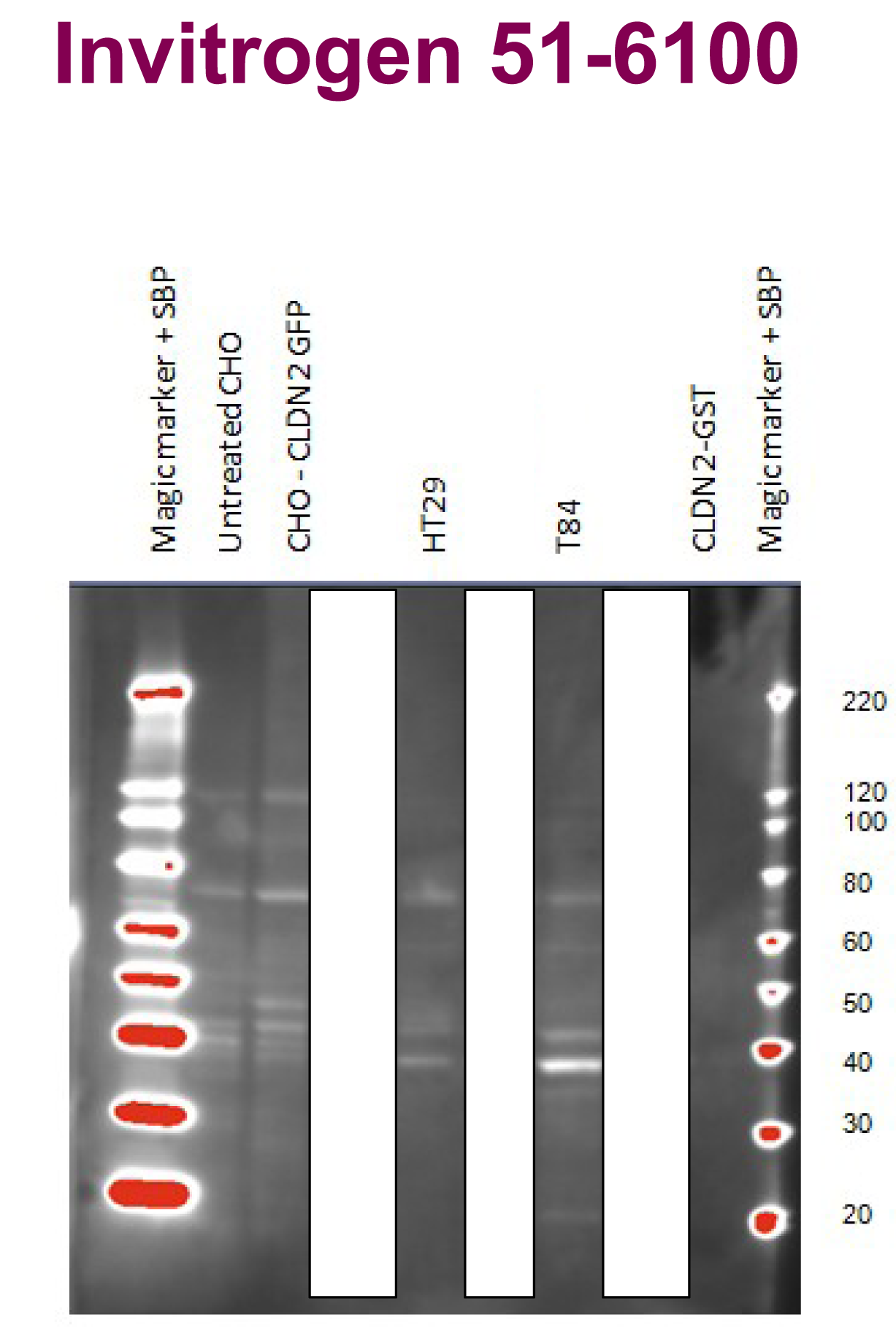

Supplement: S9 Fig — Western blot of equally loaded cell lysates and recombinant CLDN2-GST protein was probed with Invitrogen 51–6100 anti-CLDN2 antibody. Antibody failed to recognise recombinant protein, and picked up a ladder of non-specific bands in endogenously expressing HT29,T84. 51–6100 did not distinguish between non-transfected CHO-K1 and CHO-K1 cells overexpressing CLDN2-GFP protein. (TIF) [file pone.0162076.s009.tif]
